# Bone marrow microenvironments that contribute to patient outcomes in newly diagnosed multiple myeloma: A cohort study of patients in the Total Therapy clinical trials

**DOI:** 10.1371/journal.pmed.1003323

**Published:** 2020-11-04

**Authors:** Samuel A. Danziger, Mark McConnell, Jake Gockley, Mary H. Young, Adam Rosenthal, Frank Schmitz, David J. Reiss, Phil Farmer, Daisy V. Alapat, Amrit Singh, Cody Ashby, Michael Bauer, Yan Ren, Kelsie Smith, Suzana S. Couto, Frits van Rhee, Faith Davies, Maurizio Zangari, Nathan Petty, Robert Z. Orlowski, Madhav V. Dhodapkar, Wilbert B. Copeland, Brian Fox, Antje Hoering, Alison Fitch, Katie Newhall, Bart Barlogie, Matthew W. B. Trotter, Robert M. Hershberg, Brian A. Walker, Andrew P. Dervan, Alexander V. Ratushny, Gareth J. Morgan

**Affiliations:** 1 Bristol Myers Squibb, Seattle, Washington, United States of America; 2 Sage Bionetworks, Seattle, Washington, United States of America; 3 Cancer Research and Biostatistics, Seattle, Washington, United States of America; 4 Myeloma Center, University of Arkansas for Medical Sciences, Little Rock, Arkansas, United States of America; 5 Genmab, Princeton, New Jersey, United States; 6 The University of Texas MD Anderson Cancer Center, Houston, Texas, United States of America; 7 Winship Cancer Institute, Emory University, Atlanta, Georgia, United States of America; 8 Department of Hematology and Medical Oncology, Tisch Cancer Institute, Icahn School of Medicine at Mount Sinai, New York, New York, United States of America; 9 BMS Center for Innovation and Translational Research Europe (CITRE), Seville, Spain; Kumamoto University Hospital, JAPAN

## Abstract

**Background:**

The tumor microenvironment (TME) is increasingly appreciated as an important determinant of cancer outcome, including in multiple myeloma (MM). However, most myeloma microenvironment studies have been based on bone marrow (BM) aspirates, which often do not fully reflect the cellular content of BM tissue itself. To address this limitation in myeloma research, we systematically characterized the whole bone marrow (WBM) microenvironment during premalignant, baseline, on treatment, and post-treatment phases.

**Methods and findings:**

Between 2004 and 2019, 998 BM samples were taken from 436 patients with newly diagnosed MM (NDMM) at the University of Arkansas for Medical Sciences in Little Rock, Arkansas, United States of America. These patients were 61% male and 39% female, 89% White, 8% Black, and 3% other/refused, with a mean age of 58 years. Using WBM and matched cluster of differentiation (CD)138-selected tumor gene expression to control for tumor burden, we identified a subgroup of patients with an adverse TME associated with 17 fewer months of progression-free survival (PFS) (95% confidence interval [CI] 5–29, 49–69 versus 70–82 months, χ^2^ *p* = 0.001) and 15 fewer months of overall survival (OS; 95% CI –1 to 31, 92–120 versus 113–129 months, χ^2^ *p* = 0.036). Using immunohistochemistry-validated computational tools that identify distinct cell types from bulk gene expression, we showed that the adverse outcome was correlated with elevated CD8^+^ T cell and reduced granulocytic cell proportions. This microenvironment develops during the progression of premalignant to malignant disease and becomes less prevalent after therapy, in which it is associated with improved outcomes. In patients with quantified International Staging System (ISS) stage and 70-gene Prognostic Risk Score (GEP-70) scores, taking the microenvironment into consideration would have identified an additional 40 out of 290 patients (14%, premutation *p* = 0.001) with significantly worse outcomes (PFS, 95% CI 6–36, 49–73 versus 74–90 months) who were not identified by existing clinical (ISS stage III) and tumor (GEP-70) criteria as high risk. The main limitations of this study are that it relies on computationally identified cell types and that patients were treated with thalidomide rather than current therapies.

**Conclusions:**

In this study, we observe that granulocyte signatures in the MM TME contribute to a more accurate prognosis. This implies that future researchers and clinicians treating patients should quantify TME components, in particular monocytes and granulocytes, which are often ignored in microenvironment studies.

## Author summary

**Why was this study done?:**

- The cells around a tumor, also known as the tumor microenvironment (TME), can help a tumor grow by suppressing the immune system or fight a tumor by mounting an immune response.
- Most studies of multiple myeloma (MM) have focused on the tumor itself, rather than the bone marrow (BM) TME in which the tumor is growing.
- We hypothesized that the MM TME held clues that could help us better treat patients.

**What did the researchers do and find?:**

- We used a gene-expression–based computational technique to determine which cell types were present in patient BM.
- Patients with BM lacking a family of innate immune cells called granulocytes presented with worse outcomes compared to other patients.
- As MM progresses from a predisease to a cancerous state, the percentage of granulocytes decreases; the patients with the fewest granulocytes had more serious diseases.

**What do these findings mean?:**

- If granulocytes help myeloma patients respond to therapy, then addressing the decline in granulocytes may improve MM treatment.
- Patients with MM and few granulocytes in their BM should be watched for worse outcomes.

## Introduction

A major aim of modern cancer therapy is to develop precision immuno-oncology in which treatment is targeted to the biology of distinct disease segments [1]. Multiple myeloma (MM) is a bone marrow (BM) cancer for which modern therapy has increased 5-year patient survival from approximately 35% in 2000 to nearly 70% in 2017 [2]; however, more than 10% of patients do not respond well [3], necessitating novel treatment modalities. To date, work on disease stratification in MM has focused on the clonal plasma cells (the tumor) rather than the environment (the BM) in which the tumor proliferates. This work has led directly to the translocation cyclin D classification [4–6]; however, tumor-mutation–based stratification does not fully explain patient outcomes. Furthermore, these approaches have failed to identify a consistent mutational pattern driving disease progression from abnormal, but premalignant, plasma cells to cancer.

MM often starts as monoclonal gammopathy of undetermined significance (MGUS), in which a clonal plasma cell has multiplied to constitute <10% of the plasma cells in the BM but the patient is otherwise asymptomatic. Each year, about 1% of patients with MGUS will progress to MM. Should that clonal plasma cell proliferate to account for >10% of plasma cells, the patient is considered to have asymptomatic or smoldering multiple myeloma (SMM). About 10% of patients with SMM will progress to MM each year [7]. Attempts to define the nature of the plasma cell mutations driving disease progression from MGUS through SMM to MM requiring therapy have largely failed and instead focused attention on changes in subclonal structure driven potentially by the tumor microenvironment (TME) [8].

**Fig 1:**
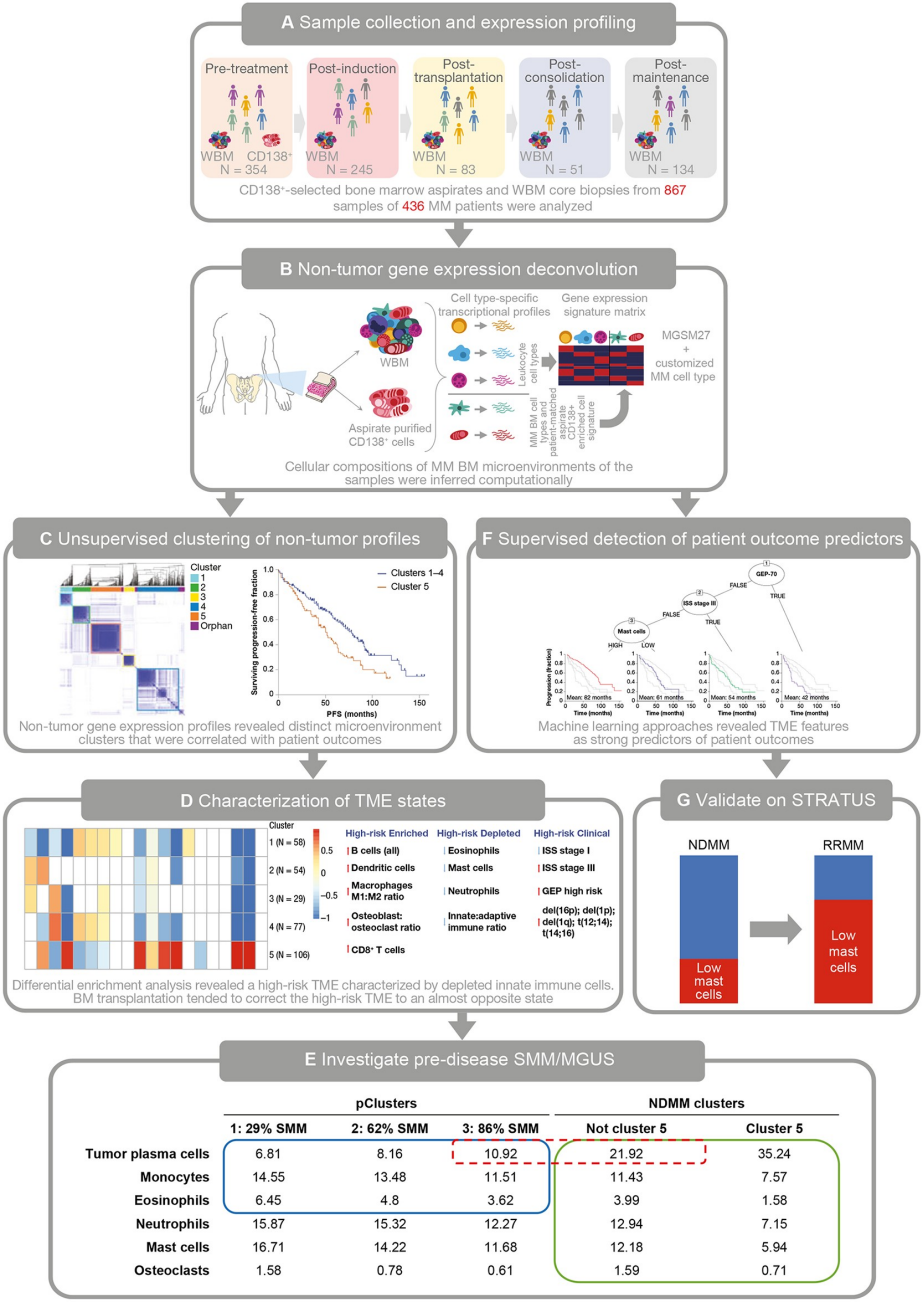
On-treatment MM project overview. (A) Samples from 436 patients with NDMM were taken during the course of treatment, resulting in 867 total samples. (B) The putatively pure tumor CD138^+^ samples were combined with the LM22 leukocyte signature matrix and 5 additional BM-specific cell types to determine the cell-type mix in the WBM samples. Illustration based on [18]. (C) The proportion of gene expression from the tumor was computationally removed from the WBM samples, which were then assigned to one of the 5 unique microenvironment clusters shown on the left side of the panel. One of these clusters (termed “low-granulocyte” Cluster 5) was associated with lower patient PFS/OS as shown on the right side of the panel. (D) Cluster 5 had a distinct microenvironment characterized by unusual levels of various cell types. (E) Follow-up studies on predisease SMM and MGUS patients revealed that this microenvironment developed as the disease progressed. As the 3 pClusters on the left contain an ever-greater portion of patients with SMM, they come to resemble the microenvironment for Cluster 1–4 patients with NDMM. Cluster 5 NDMM patients show an extension of the trends observed as the disease develops. (F) Machine learning revealed that patients with low levels of granulocytes had consistently worse outcomes, even after accounting for high-risk status using GEP-70 and ISS stage. (G) As predicted, patients with RRMM in the STRATUS trial were significantly enriched for the low-granulocyte phenotype. BM, bone marrow; CD, cluster of differentiation; del, deletion; GEP-70, 70-gene Prognostic Risk Score; IMiD, immunomodulatory drug; ISS, International Staging System; LM22, leukocyte matrix 22; MGSM, myeloma genome signature matrix; MGUS, monoclonal gammopathy of undetermined significance; MM, multiple myeloma; NDMM, newly diagnosed multiple myeloma; OS, overall survival; pCluster, premalignant microenvironment cluster; PFS, progression-free survival; RRMM, relapsed/refractory multiple myeloma; SMM, smoldering multiple myeloma; STRATUS, Evaluation of Safety of Pomalidomide in Combination With Dexamethasone (Low Dose) in Patients With Refractory or Relapsed and Refractory Multiple Myeloma; t, translocation; TME, tumor microenvironment; UAMS70, University of Arkansas for Medical Sciences 70-gene signature; WBM, whole bone marrow.

The importance of microenvironmental interactions between clonal plasma cell and stromal cells mediated by autocrine and paracrine loops has been appreciated for some time [9–11]. However, little information is available on quantifying the global pathologic impact of the clone on the microenvironment [12,13]. Previous work has addressed the impact of clonal tumor cells on T cell subsets, natural killer (NK) cells, macrophages, dendritic cells, and high-density neutrophils [4–6,8–10], but none of these studies took into account the contextual composition of nontumor cells within the whole bone marrow (WBM). We hypothesized that understanding the TME will improve our ability to identify patients who will have worse outcomes with current therapies and point toward potential new treatment avenues for those patients. This hypothesis was validated using the experimental design shown in Fig 1.

## Methods

### Patient data

Patients considered for analysis were enrolled into the Total Therapy (TT) 3–5 trial series; details of these protocols have been described in previous publications [14–16]. These patients were enrolled between February 25, 2004 and January 11, 2014 with 25–5,241 days of follow-up (mean 2,628 days, standard deviation 1,387 days, S1–S3 Tables). They included 266 males and 170 females aged 32.4–75.2 years at baseline (mean = 58.48, median = 59.85); 387 identified as White, 34 as Black, 5 as Hispanic, 5 as Asian, 3 as Native American, and 2 refused to identify a race. TT 3–5 generally includes treatment with thalidomide, dexamethasone, and autologous BM transplant with personalized treatment regimens informed by tumor gene expression and cytogenetics. In this study, samples were grouped by treatment stage into time points: pretreatment, postinduction, post-transplantation, postconsolidation, and postmaintenance (Fig 1A). All TT protocols were approved by the Institutional Review Board of the University of Arkansas for Medical Sciences. All patients provided written informed consent prior to enrollment in keeping with institutional, federal, and international guidelines. All patients enrolled in TT 3–5 with at least one WBM biopsy sample taken prior to the initiation of therapy were considered for analysis. All patients provided written informed consent to approve the use of their BM biopsy samples for research in accordance with the Declaration of Helsinki of 2013. Additional patient and sample data are provided in S1–S3 Tables.

### Ethics statement

Previously untreated patients with newly diagnosed multiple myeloma (NDMM) recruited to the TT clinical trials between February 2004 and August 2017 were included in this investigation after written informed consent. The sample collection protocol was approved by the University of Arkansas for Medical Sciences Institutional Review Board (protocol #2012–12). This study was performed in accordance with the 1964 Declaration of Helsinki. Ethics approval was not required for this analysis. This study has been reported according to the STROBE guideline available in S1 Text, STROBE checklist.

### Methods overview

Analysis of RNA extracted from WBM biopsies potentially contains information necessary to provide a comprehensive analysis of the microenvironment [17]. To extract this information, we developed a custom algorithm able to define and quantitate the cellular subcomponents of the BM microenvironment. We applied it to WBM trephine biopsies taken from the aforementioned TT patients. These biopsies were paired with pathologist-estimated tumor fractions and cluster of differentiation (CD)138^+^-selected plasma cells from those patients, allowing the signal from the malignant plasma cells to be removed and the focus put on the residual signal derived from the microenvironment.

### Generation of myeloma genome signature matrix 27 (MGSM27)

To deconvolve the cellular content of the BM of plasma cell disorders, we upgraded a pre-existing 22-leukocyte signature matrix [18] by adding tumor and 4 BM cell types (S1A Fig). Deconvolution of samples purified to contain only single cell types revealed cell types that are likely to be mis-deconvolved as other cell types (S1B Fig). This spillover, combined with biological considerations, led to post hoc combination of several deconvolution estimates, resulting in the 18 cell types presented in this analysis. More details about signature matrix construction, including a usable version of MGSM27, are available in the Automated Deconvolution Augmentation of Profiles for Tissue Specific cells [19] package available on the Comprehensive R Archive Network (https://cran.r-project.org/web/packages/ADAPTS/index.html).

### Testing deconvolution cell-type predictions

The digital cell quantifier algorithm [20] was modified to deconvolve cell types from bulk mRNA expression data using our custom MGSM27 signature matrix. DCQ was chosen because it outperformed other deconvolution methods in our internal trials (S1H and S1I Fig). To ensure that the algorithm could accurately estimate malignant plasma cells and other cell types from bulk gene expression, we calibrated CD138^+^ cell-type estimates (normal and malignant plasma cells) against clinical estimates of myeloma plasma cell percentage in WBM (immunohistochemistry and flow cytometry) and purified CD138^+^ aspirates. The deconvolution method was then validated against publicly available PBMC data [21] and pathologist estimates of eosinophils (S1E Fig, S4 Table), neutrophils (S1F Fig, S5 Table), and mast cells (S6 Table) in subsets of our patient samples.

### Clustering of BM based on its microenvironment

**Fig 2:**
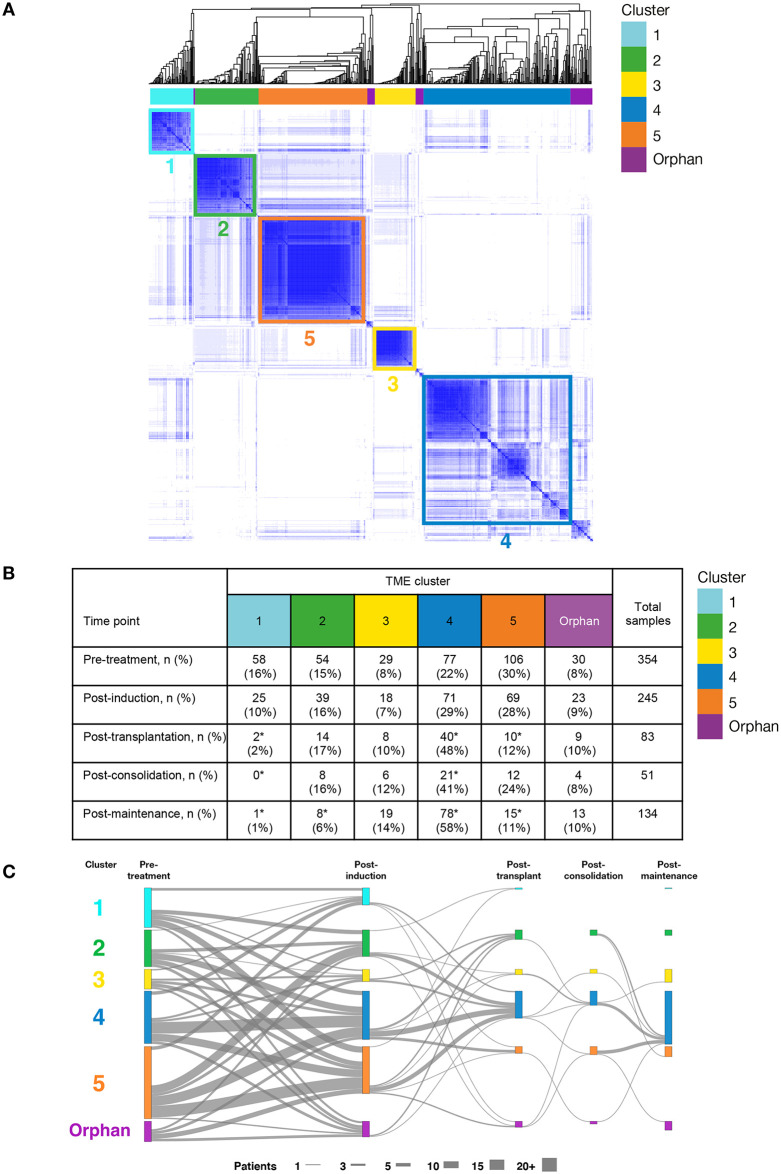
Pseudo-CD138^–^ gene-expression clusters: Clustering of 867 samples with tumor signature removed based on deconvolution. (A) Columns show the 867 patient samples; rows show the high-variance genes, with relatively low expression shown in white and high expression shown in blue. Hierarchical clustering and Bayesian information criteria analysis revealed 5 large clusters, with all other samples (shown in purple) classed as orphan clusters. (B) Cluster membership is distributed across treatment time points. (C) A Sankey diagram showing the cluster population shifts that occur during treatment. Colored lines are scaled to show the number of patients in each cluster at each time point. Gray lines indicate transitions between time points. Portions of colored lines with no attached gray lines had no samples in the immediately preceding or following time point. *Proportions significantly different to pretreatment time point (i.e., binomial false discovery rate < 0.05). CD, cluster of differentiation; TME, tumor microenvironment.

To address whether there were distinct clusters within the microenvironment of NDMM, we constructed pseudo-CD138^–^ gene-expression profiles from the WBM by removing the signature of CD138^+^ cells. Patient samples from all time points (N = 867) were clustered using unsupervised machine learning to identify groups of patients with similar BM microenvironments. Fig 2A shows how patients in each cluster have similar BM gene-expression patterns. Fig 2B and 2C shows the number of patients in each of those clusters and how they change during the course of treatment.

### Statistical analyses

For supervised machine-learning survival analysis, cell-type estimates for cells with similar functions and gene-expression signatures were added together to create 19 cell types for assessment (S1B Fig). To determine which combinations of cell types and cytogenetic characteristics best separated patients with relative long and short overall survival (OS) and progression-free survival (PFS), we used conditional inference trees [22] with the Cox proportional hazards model [23], which conveniently included permutation tests to help determine generalizability.

Kaplan–Meier curves were generated using the R Survival package [24], and *p*-values were estimated with a χ^2^ test. Estimated survival times were calculated by the *survfit* function and generally reported as restricted mean ± 1 standard error. Regression analysis (including lasso regression) to evaluate feature performance was performed using Cox regression in the R package *GLMnet* (Bioconductor) [24,25]. Decision trees were generated using the conditional inference trees algorithm, which accounts for censoring using a Cox proportional hazards model and only adds variables when they pass a *p*-value cutoff as calculated by a permutation test [22,26].

Stepwise Cox regression was performed by first selecting the variable with the lowest univariate *p*-value. At each subsequent step, variables were evaluated with an *F* test to determine which had the lowest *p*-value. If that *p*-value was < 0.1, then that variable was added to the model. A *t* test was then performed on the regression coefficients, and any variable (except age and sex) was removed if its coefficient *p*-value was >0.05. The procedure was continued until no more variables could be added to the model. To prevent bias in statistical analysis, results were calculated in parallel by 2 teams. One focused on lasso regression, which features predominantly in machine-learning literature; the other used stepwise regression, a technique rooted in conservative clinical and statistical analysis. Differences in these analyses (e.g., quartiles versus continuous regression) arose as a consequence of this independent verification.

Additional methods are available in S2 Text, supplementary methods.

## Results

### Validation of MGSM27 cell-type predictions

The MGSM27 signature matrix correctly identified that, post-CD138^+^ selection, the samples were almost entirely pure CD138^+^ cells (N = 423; Wilcoxon *p* < 0.001; S1C Fig). In WBM samples in which the global RNA contribution of the CD138^+^ cells to the admixture was heterogeneous (S1D Fig), the correlation coefficient was 0.63 with a root mean-square error (RMSE) of 9.99% (N = 247; Fisher’s Z *p* < 0.001). The predictions also mirrored pathologist estimates of eosinophil and neutrophil numbers. Eosinophils had a correlation coefficient of 0.27 with an RMSE of 3.05% (N = 345; Fisher’s Z *p* < 0.001; S1E Fig), and neutrophils had a correlation coefficient of 0.32 with an RMSE of 9.55% (N = 356; Fisher’s Z *p* ≤ 0.001; S1F Fig).

Follow-up studies stained a small number of samples (n = 20) with the mast cell marker CD117 (c-Kit). CD117^+^ cells with morphological characteristics of immature mast cells were correlated with deconvolved mast cell predictions (Pearson’s *r* = 0.319, Fisher’s Z *p* = 0.171, n = 20) and also with neutrophils (*r* = 0.449, *p* = 0.047, n = 20) and monocytes (*r* = 0.489, *p* = 0.029, n = 20), as well as other cell types (S6E Fig). Morphologically mature CD117^+^ mast cells were unexpectedly rare (much less than the previously reported 4.8% of cells in active myeloma) [27,28], and these CD117^+^ cells did not correlate with deconvolved mast cell estimates.

The MGSM 27 signature matrix was also applied to publicly available peripheral blood mononuclear gene-expression data (E-MTAB-3732) [21]. This test of the algorithm’s ability to reconstruct all components of mixed cell-type samples revealed that the estimated cell percentages fell within the expected clinical ranges (S1G Fig).

### BM microenvironment clusters

**Fig 3:**
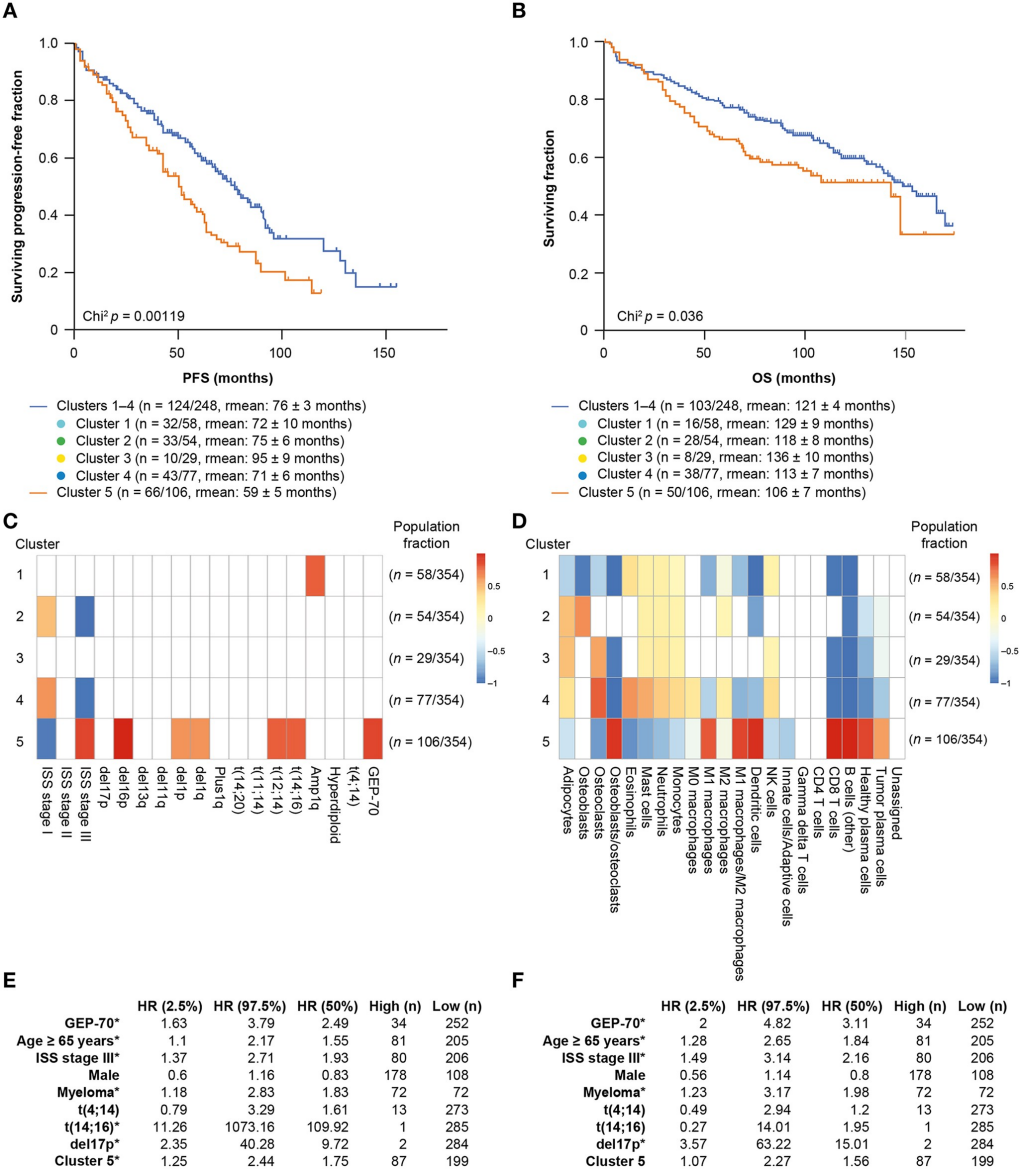
Clinical outcomes and cellular components of microenvironment clusters: Kaplan–Meier curves for pretreatment sample modeling. (A) PFS for the “low-granulocyte” Cluster 5 (orange; n = 66/106; rmean: 59 ± 5 months) versus Clusters 1–4 (blue; n = 124/248; rmean = 76 ± 3 months). (B) OS for the “low-granulocyte” Cluster 5 (orange; n = 50/106; rmean: 106 ± 7 months) versus Clusters 1–4 (blue; n = 103/248; rmean = 121 ± 4 months). (C) Average cytogenetic and clinical characteristic enrichment for patients in each of the 5 clusters. (D) Average deconvolved tumor and stromal cell types present in each of the 5 clusters. FDRs were calculated using Wilcoxon signed-rank test and Benjamini–Hochberg correction. FDRs > 0.05 are white. (E, F) Univariate Cox proportional HRs for PFS and OS (respectively). The analysis considered the upper quartile versus the lowest quartile (or high versus low if binary) for variables in pretreatment samples. Those cell types, ratios, and clinical characteristics marked with an asterisk remain after multivariate Cox proportional hazards lasso regression, implying that they contain independent information about patient outcomes. CD, cluster of differentiation; del, deletion; FDR, false discovery rate; GEP-70, 70-gene Prognostic Risk Score; HR, hazard ratio; ISS, International Staging System; NK, natural killer; OS, overall survival; PFS, progression-free survival; t, translocation.

Clustering identified 5 main microenvironment clusters (Fig 2A), with patient demographics outlined in Table 1. Of these clusters, Cluster 5 was unique both in terms of immune composition and patient outcomes (Fig 3 and S2 Fig).

**Table 1:** Baseline patient demographics.

Factor, n/N (%)	All patients	Cluster 1	Cluster 2	Cluster 3	Cluster 4	Cluster 5	Orphan	*p*-Value^a^
Age ≥ 65 years	104/354 (29)	18/59 (31)	17/54 (31)	3/28 (11)	24/76 (32)	32/107 (30)	10/30 (33)	0.284
Female	137/354 (39)	21/59 (36)	13/54 (24)	10/28 (36)	32/76 (42)	53/107 (50)	8/30 (27)	0.023
White	314/354 (89)	54/59 (92)	50/54 (93)	27/28 (96)	67/76 (88)	90/107 (84)	26/30 (87)	0.326
ISS Stage 1	130/354 (37)	17/59 (29)	26/54 (48)	12/28 (43)	44/76 (58)	17/107 (16)	14/30 (47)	< 0.001
ISS Stage 2	132/354 (37)	23/59 (39)	22/54 (41)	11/28 (39)	22/76 (29)	45/107 (42)	9/30 (30)	0.485
ISS Stage 3	92/354 (26)	19/59 (32)	6/54 (11)	5/28 (18)	10/76 (13)	45/107 (42)	7/30 (23)	< 0.001
Albumin < 3.5 g/dL	124/354 (35)	23/59 (39)	13/54 (24)	9/28 (32)	20/76 (26)	49/107 (46)	10/30 (33)	0.043
B2M ≥ 3.5 mg/L	183/354 (52)	35/59 (59)	21/54 (39)	12/28 (43)	21/76 (28)	81/107 (76)	13/30 (43)	< 0.001
B2M > 5.5 mg/L	92/354 (26)	19/59 (32)	6/54 (11)	5/28 (18)	10/76 (13)	45/107 (42)	7/30 (23)	< 0.001
Creatinine ≥ 2 mg/dL	16/354 (5)	5/59 (8)	3/54 (6)	1/28 (4)	2/76 (3)	4/107 (4)	1/30 (3)	*
Hb < 10 g/dL	134/354 (38)	24/59 (41)	12/54 (22)	6/28 (21)	14/76 (18)	68/107 (64)	10/30 (33)	< 0.001
LDH ≥ 190 U/L	73/354 (21)	8/59 (14)	10/54 (19)	5/28 (18)	12/76 (16)	31/107 (29)	7/30 (23)	0.171
Cytogenetic abnormalities	131/345 (38)	17/58 (29)	18/52 (35)	8/27 (30)	22/74 (30)	53/104 (51)	13/30 (43)	0.026

n, number with factor; N, number with valid data for factor.

*Sample size assumption for the χ^2^ test is not met.

^a^*p*-values represent a comparison between groups, not against the overall population.

**Abbreviations**: B2M, beta-2 microglobulin; Hb, hemoglobin; ISS, International Staging System; LDH, lactate dehydrogenase.

Cell-type estimates from the WBM revealed that Cluster 5 was also noteworthy for its distinct microenvironment. It was significantly enriched (false discovery rate [FDR] ≤ 0.05) for estimated T cells, dendritic cells, M1 macrophages, tumor plasma cells, and other B cells (Fig 3C and 3D) and was significantly depleted for adipocytes, osteoclasts, eosinophils, mast cells, neutrophils, monocytes, M0 and M2 macrophages, and NK cells, with a lower innate/adaptive immune cell ratio. It was also enriched for International Staging System (ISS) stage III samples, as well as signs of a more malignant tumor: 70-gene Prognostic Risk Score (GEP-70) high risk, deletion (del)16p, translocation (t)(12;14), and t(14;16) (Fig 3C).

The “high-granulocyte” Cluster 4 was the most different from Cluster 5 (Fig 3C and 3D): it had the highest number of eosinophils, mast cells, neutrophils, monocytes, M0 macrophages, and osteoclasts, and it was also enriched for ISS stage I samples. Cluster 1 was enriched for amplification of 1q, a high-risk factor [29], and showed a lack of BM resident cells, such as adipocytes, osteoblasts, osteoclasts, and inflammatory cells such as M1 macrophages and dendritic cells. However, this cluster had standard PFS/OS times and was effectively eradicated post-transplantation. Clusters 2 and 3 showed similar levels of adipocytes, innate cells, and lymphocytes, but they had inverse ratios of osteoblasts and osteoclasts. Whereas Cluster 2 had an M2 macrophage signal, Cluster 3 had an increase in the NK cell population with modest positive outcomes (estimated mean pretreatment PFS was 95 ± 9 months, 10 events, χ^2^ *p* = 0.087 [S2A Fig], and OS was 136 ± 10 months, 8 events, χ^2^ *p* = 0.082; n = 29 patients; S2B Fig).

### Cluster 5 showed a negative impact on survival

Microenvironment Cluster 5 had significantly impaired mean PFS of 59 ± 5 months (66 events, n = 106) compared with 76 ± 3 months (124 events, n = 248) for the other clusters combined (χ^2^ *p* = 0.001; Fig 3A); the mean OS was 106 ± 7 months (50 events, n = 106) compared with 121 ± 4 months (103 events, n = 248) for the other clusters (χ^2^ *P* = 0.036; Fig 3B). As might be expected, Cluster 5 was significantly enriched for the level of malignant plasma cell infiltration (Fig 3D); however, the cluster persisted even when the tumor load was reduced by therapy (Fig 2B) and so was not driven by it. The adverse outcome of Cluster 5 was an independent prognostic feature in multivariate analysis including percentage myeloma infiltration, GEP-70, ISS stage III status, high-risk cytogenetics, age, sex, and Cluster 5 membership to predict OS and PFS using Cox lasso regression [30]. Even in the presence of these variables, Cluster 5 was still significantly associated with adverse PFS, but not OS (Fig 3E and 3F).

### Microenvironment cluster status was modified by treatment

Microenvironment clusters revealed patient dynamics in 4 settings related to treatment: postinduction (N = 245 individual patient samples available), post-transplantation (n = 83), postconsolidation (n = 51), and postmaintenance (n = 134) (Fig 2B). Although the fraction of patients in each cluster changed during treatment, no new clusters emerged (Fig 2B). The “high-granulocyte” Cluster 4 was the second most frequent subtype at diagnosis after “low-granulocyte” Cluster 5 and became the most common TME cluster after transplantation, constituting 41%–58% of patients post-transplantation (Fig 2B). Other smaller clusters showed different dynamics; e.g., “low-osteoblast” Cluster 1 decreased from 16% in the pretreatment patient population to 2% after transplantation (Fig 2B).

Finally, “low-granulocyte” Cluster 5 decreased from 30% pretreatment to 28% postinduction to 12% after transplantation. Fig 2C shows the patient cluster transitions that occurred between the pre- and post-transplantation phases. Of the 63 patients for whom we have both pretreatment and post-transplantation paired samples, 18 of 20 Cluster 5 patients (defined by their pretreatment cluster membership) moved into other TME clusters, with 13 moving into “high-granulocyte” Cluster 4. Of note, the 69 (28%) of patients in Cluster 5 at the postinduction time point exhibited significantly worse outcomes when compared with patients whose postinduction TME was not in Cluster 5 (176 [72%] patients; S2 Fig); estimated mean PFS was 57 ± 6 months (45 events, n = 69) for Cluster 5 compared with 76 ± 4 months (83 events, n = 176) for Clusters 1–4 (χ^2^ *p* = 0.004; S2C Fig); estimated mean OS was 101 ± 8 months (37 events, n = 69) for Cluster 5 compared with 127 ± 5 months (62 events, n = 176) for Clusters 1−4, χ^2^ *p* = 0.002; S2D Fig). Patients who started in Cluster 5 and stayed there did not have significantly different PFS or OS than those who left.

When patient samples taken at relapse (within 180 days prior to clinically defined relapse or up to 30 days after relapse, n = 17) were compared with samples taken during complete remission (n = 108), all 8 of the significantly different (*p* ≤ 0.05) TME components (adipocytes, eosinophils, healthy plasma cells, mast cells, monocytes, neutrophils, osteoclasts, and tumor plasma cells; S3 Fig) were significantly different in and concordant with the “low-granulocyte” Cluster 5. In particular, estimated granulocytes were significantly lower for patients who were about to relapse. Taken together, this implies a diverse array of initial MM microenvironment states that tend to respond to treatment and converge on a “high-granulocyte” Cluster 4 remission state. In contrast, the subset of patients who retain a “low-granulocyte” Cluster 5 microenvironmental state had impaired outcomes.

### The cellular content of the high-risk microenvironment was associated with outcome

**Fig 4:**
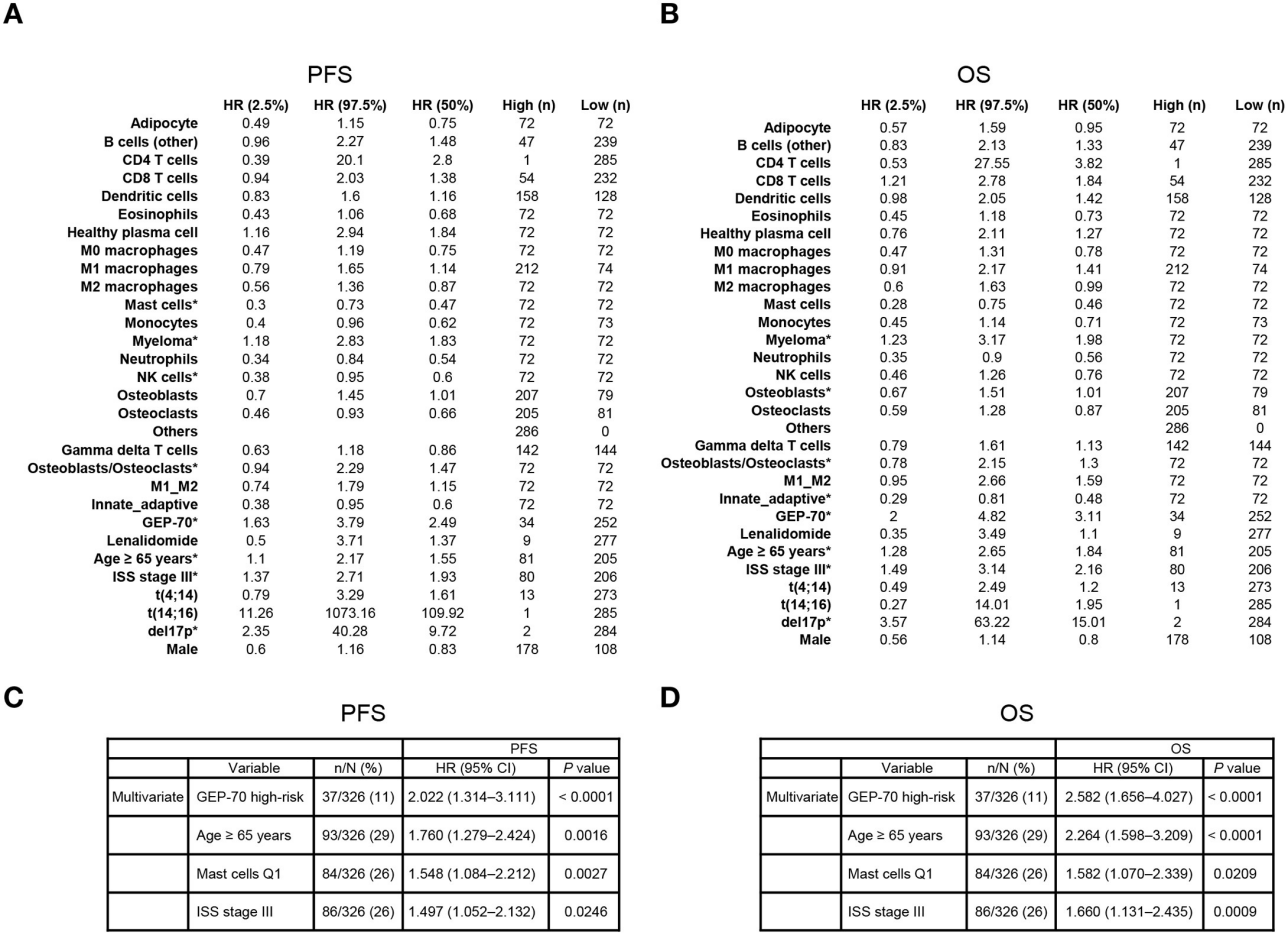
Pretreatment regression models for cell-type, clinical, and cytogenetic characteristics. (A, B) Univariate Cox proportional HRs for PFS and OS (respectively) from 286 pretreatment samples with full cytogenetics and tumor burden data. Those cell types, ratios, and cytogenetic and clinical characteristics marked with an asterisk remain after multivariate Cox proportional hazard lasso regression, implying that they contain independent information about patient outcomes. Myeloma refers to the pathologist estimates of tumor cell percentages (rather than the deconvolution estimates). Lenalidomide indicates those 9 patients who received lenalidomide during treatment, testing whether this was skewing the results. (C, D) Variables added during stepwise Cox regressing using variable quartiles as predictors. CD, cluster of differentiation; CI, confidence interval; del, deletion; GEP-70, 70-gene Prognostic Risk Score; HR, hazard ratio; ISS, International Staging System; NK, natural killer; OS, overall survival; PFS, progression-free survival; Q, quartile; t, translocation.

The granulocytes and other depleted cell types typical of Cluster 5 were strong univariate predictors of both poor OS and PFS across all 354 patients with NDMM (Fig 4A and 4B); e.g., eosinophils (OS, log-rank *p* = 0.02 PFS, log-rank *p* = 0.05), mast cells (OS, log-rank *p* = 0.001; PFS, log-rank *p* = 0.003), monocytes (OS, log-rank *p* = 0.006; PFS, log-rank *p* = 0.006), and neutrophils (OS, log-rank *p* < 0.001; PFS, log-rank *p* = 0.01) (S4 Fig). This is concordant with the high correlation between the counts of the myeloid lineage cell types: mast cells are strongly correlated with eosinophils (Pearson’s *r* = 0.829), neutrophils (*r* = 0.828), and monocytes (*r* = 0.830). We independently verified the granulocyte signature using biclusters [31] constructed by cMonkey2 [32–34] and functional enrichment analysis using the Gene Ontology Biological Process terms (S5 Fig) [35,36]. Biclusters significantly enriched for granulocyte-annotated genes were also positively correlated with PFS.

Cox lasso regression determined that TME features had not been confounded by tumor burden or other known high-risk features. Regression models including CD138^+^ percentage, ISS stage, GEP-70 risk status, age ≥ 65 years, high-risk cytogenetics, or sex tested whether cellular subtypes would be removed from the model. These models, for both PFS and OS, retained tumor burden, mast cells, osteoblast/osteoclast ratio, GEP-70, ISS stage III, del17p, and age ≥ 65 years as significant predictors of patient outcomes (Fig 4A and 4B).

Stepwise Cox regression similarly demonstrated that granulocytes retained predictive power after correcting for known prognostic markers. The stepwise regression selected GEP-70 high risk, age ≥ 65 years, low mast cells (quartile 1 versus other), and ISS stage III as the most predictive factors for PFS and OS (Fig 4C and 4D). Thus, the deconvolved mast cell counts add significant information beyond that provided by GEP-70 and ISS stage.

Immunohistochemistry-estimated granulocyte percentages were also predictive of patient outcomes. Conditional inference decision trees [22,26] built for the 290 patients with immunohistochemistry data (S6A–S6D Fig) revealed that low-granulocyte percentages were associated with adverse outcomes; e.g., low eosinophil counts as determined by immunohistochemistry were a significant predictor of outcome (permutation *p* < 0.001 for PFS; permutation *p* = 0.045 for OS) with a mean PFS of 67 ± 4 months (115 events, N = 195) and OS of 112 ± 5 months (90 events, N = 195) compared with a mean PFS of 97 ± 10 months (13 events, N = 31) and OS of 131 ± 6 months (34 events, N = 95) for patients with high counts.

**Fig 5:**
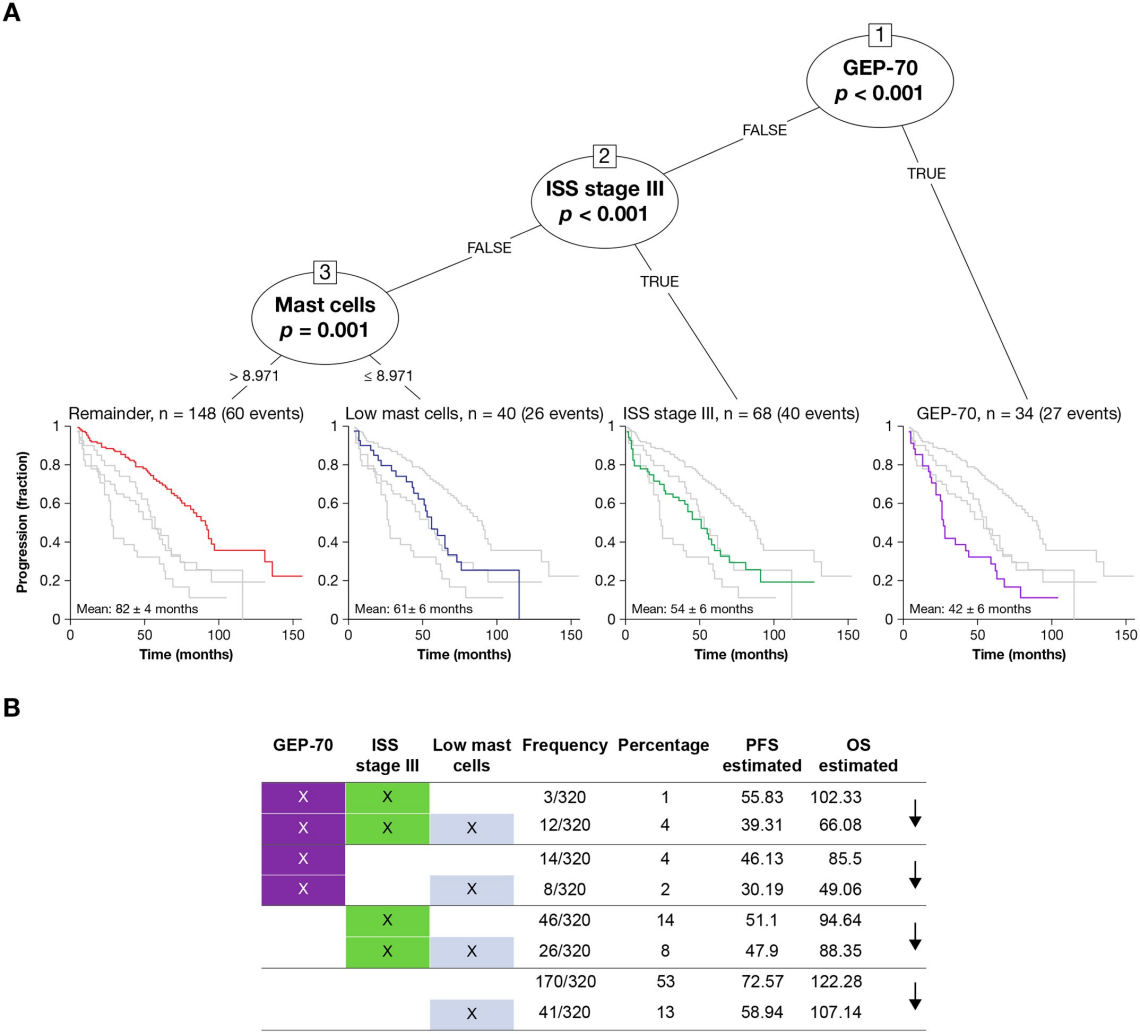
Mast cells, patient outcomes, and TME. (A) Cox proportional hazards conditional inference tree that selected an optimal combination of attributes from the cell types and clinical characteristics for the 290 pretreatment samples with known cytogenetics and tumor burden estimates. The algorithm first selects the strongest attribute (the cell-type or clinical characteristic with the lowest *p*-value), in this case, the GEP-70 high-risk flag (*p* < 0.001). As expected, those 34 patients (27 of whom had an event) with GEP-70 high-risk tumors have particularly bad outcomes (estimated mean PFS = 42 months), and no other attribute can significantly split the patient population. The algorithm then selects the attribute that best divides the remaining patient population, which is ISS stage III status (*p* < 0.001). These 68 patients (40 of whom had an event) without GEP-70 high-risk tumors but who are ISS stage III cannot be significantly divided by any other attribute and have relatively poor outcomes (estimated mean PFS = 54 months). The remaining 188 patients (86 of whom had an event) are best divided by deconvolution-based mast cell estimates (*p* = 0.001). Those 40 patients (26 of whom had an event) with predicted mast cell levels of 8.97% or lower also have relatively short PFS (estimated mean PFS = 61 months). The remaining 148 patients had a relatively long PFS of 82 months. Importantly, the model does not select any more attributes that can pass a cutoff of *p* = 0.01, implying that of these deconvolved immune cell estimates and clinical characteristics, GEP-70, ISS stage III, and mast cell estimates are the strongest predictors. Clinically estimated tumor burden, which is inversely correlated with deconvolved mast cell percentages, was not selected by the tree and is, therefore, less informative. (B) Pretreatment samples with known ISS stage, GEP-70 status, and mast cell signature scores (n = 320). The 8 rows show all possible combinations of GEP-70 high risk versus not high risk, ISS stage III versus not stage III, and mast cell signature as high versus low (≤ 8.97%). GEP-70, 70-gene Prognostic Risk Score; ISS, International Staging System; OS, overall survival; PFS, progression-free survival; TME, tumor microenvironment.

Granulocytes were important for stratifying the 286 pretreatment samples with fully quantified cytogenetics, GEP-70 high-risk status, ISS stage, and tumor burden. Conditional inference trees showed that the pretreatment samples can be best divided by GEP-70 (estimated mean PFS of 42 ± 6 months, 27 events, n = 34), ISS stage III (estimated mean PFS of 54 ± 6 months, 40 events, n = 68), and low mast cells (estimated mean PFS of 61 ± 6 months, 26 events, n = 40) compared with an estimated mean PFS of 82 ± 4 months (60 events, n = 148) for the remainder (Fig 5A). Further, patients with tumor-specific high-risk status, as defined by ISS stage or GEP-70 status, had even worse outcomes if they had low mast cell estimates (Fig 5B).

### Tumor gene-expression profiles were associated with depleted granulocytes

To address what tumor-side molecular features may be driving the identification of low mast cells and thus perhaps the poor outcomes associated with depleted granulocytes, we compared the pretransplantation gene expression of the 328 patients for whom we had paired purified CD138^+^-selected samples and WBM samples. The WBM samples were used to determine the estimated percentage of mast cells and were divided into 2 groups: those with the lowest quartile of mast cells in the WBM and the remainder with higher mast cells. The top 32 genes in the CD138^+^ RNA-expression data that were differentially expressed between these 2 groups, as measured by a Wilcoxon signed-rank test (FDR ≤ 0.05) and with an absolute log_2_ fold change > 0.66, are shown in S7 Fig.

### The development of a high-risk microenvironment during MM progression

To understand how the patterns seen in NDMM develop during the multistep progression from MGUS through SMM to NDMM, we applied the same deconvolution techniques to investigate WBM samples from 131 premalignant patients (MGUS, n = 55; SMM, n = 76). This revealed that patients with more advanced SMM have significantly increased tumor plasma cells (Wilcoxon signed-rank *p* = 0.004) and plasma cells (*p* = 0.01) and significantly decreased monocytes (*p* = 0.01) and eosinophils (*p* = 0.018), consistent with the patterns observed as patients moved toward worse outcomes in the MM cohort. Only 6 of the patients with MGUS/SMM were known to progress, so no significantly different cell types were observed. Using our previously defined 5 microenvironment clusters, 97% (127/131) of these patients (54/55 of MGUS and 73/76 of SMM) were assigned to “high-granulocyte” Cluster 4, the dominant post-transplantation remission microenvironment cluster.

**Fig 6:**
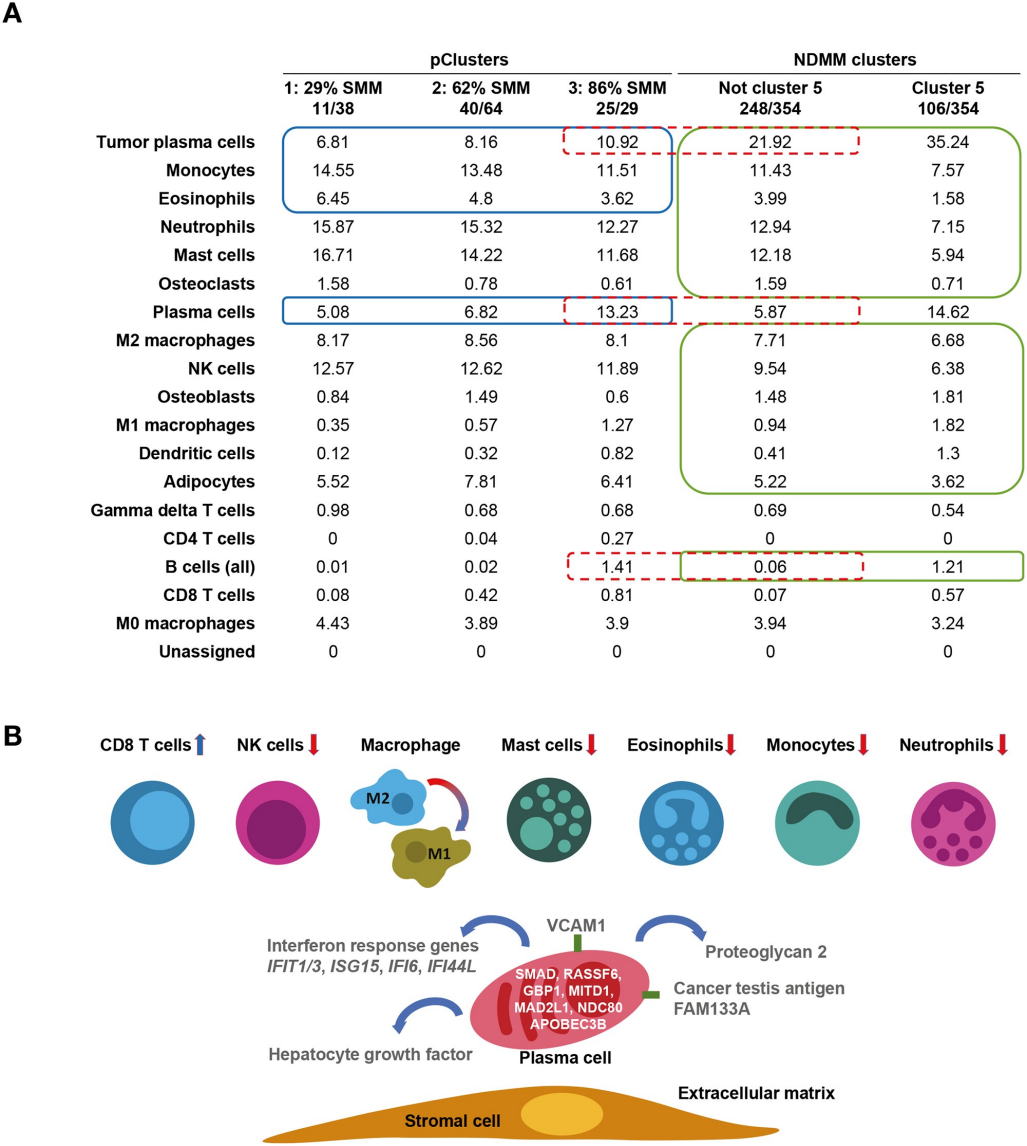
Progression of immune states through pClusters to elevated risk. (A) Shown are the deconvolved cell-type estimates for 131 samples progressing through the 3 SMM/MGUS clusters (pCluster 1: MGUS [n = 27], SMM [n = 11]; pCluster 2: MGUS [n = 24], SMM [n = 40]; pCluster 3: MGUS [n = 4], SMM [n = 25]). Also shown are 354 patients with NDMM in Cluster 5 (n = 106) and in other clusters (n = 248). The cells outlined in blue in the SMM/MGUS clusters show a significant difference (Wilcoxon FDR ≤ 0.05) between patients with SMM and those with MGUS. The cells outlined in green show a significant difference between patients with SMM and those with NDMM. The plasma cell estimates outlined in dotted red show that the overall plasma cell estimates (tumor plasma cells, plasma cells, and B cells) are very similar between SMM pCluster 3 (25.56%) and NDMM clusters that are not “low-granulocyte” Cluster 5 (27.85%). (B) Shown are the aggregate tumor and microenvironment factors that characterize the high-risk microenvironment. The top section shows cells whose proportion is elevated (blue) or reduced (red) in the high-risk microenvironment. The bottom section shows a plausible mechanism of interaction between the tumor and microenvironment involving VCAM1 and other factors shown in S7 Fig. APOBEC3B, apolipoprotein B MRNA editing enzyme catalytic subunit 3B; CD, cluster of differentiation; FAM133A, family with sequence similarity 133 member A; FDR, false discovery rate; GBP1, guanylate-binding protein 1; IFI44L, interferon-induced protein 44-like; IFI6, interferon-inducible protein 6; IFIT1/3, interferon-induced protein with tetratricopeptide repeats 1/3; ISG15, interferon-stimulated gene 15; MAD2L1, mitotic spindle assemble checkpoint protein; MGUS, monoclonal gammopathy of undetermined significance; MITD1, microtubule interacting and trafficking domain 1; NDC80, kinetochore protein NDC80 homolog; NDMM, newly diagnosed multiple myeloma; NK, natural killer; pCluster, premalignant microenvironment cluster; RASSF6, Ras association domain family member 6; SMAD, homologies to the Caenorhabditis elegans SMA ("small" worm phenotype) and Drosophila MAD ("Mothers Against Decapentaplegic") family of genes; SMM, smoldering multiple myeloma; VCAM1, vascular cell adhesion protein 1.

Unsupervised clustering of those premalignant patients, using the top 10% of genes ranked by variance, resulted in 3 premalignant microenvironment clusters (pClusters). pCluster 1 comprised 38 patients and 29% (11/38) had SMM; pCluster 2 comprised 64 patients and 63% (40/64) had SMM; pCluster 3 comprised 29 patients and 86% (25/29) had SMM. As these pClusters became enriched for SMM, plasma cell percentages increased while the estimated population of granulocytes and monocytes decreased (Fig 6A). As the disease progressed, the TME patterns became more like those of the MM patients with the worst outcomes, i.e., “low-granulocyte” Cluster 5. Notably, patients with NDMM outside Cluster 5 had profiles similar to patients in pCluster 3, including similar levels of estimated plasma cells, implying that the Cluster 5 microenvironment is the final step in disease progression (Fig 6B).

The mast cell signature remained relevant in post-treatment patients with relapsed/refractory MM (RRMM), so we explored the same patterns in 295 patients with RRMM treated with lenalidomide in the STRATUS (Evaluation of Safety of Pomalidomide in Combination With Dexamethasone (Low Dose) in Patients With Refractory or Relapsed and Refractory Multiple Myeloma) trial [37]. As may be expected for patients with poor outcomes, the patients with RRMM were enriched for a depleted mast cell signature; 70% (207/295) had low mast cell estimates compared with 27% (87/320) of baseline pretreatment patients, as shown in Fig 5B (Fisher exact *p* < 0.001).

## Discussion

Here, we present an analysis of 867 WBM samples taken during the course of treatment of 436 patients with MM (Fig 1A and 1B) and a follow-up study using samples from predisease patients (55 with MGUS and 76 with SMM, S1–S3 Tables). Purified tumor samples were used to isolate the nontumor portion of WBM gene-expression data, which clustered patients by their microenvironment signatures (Fig 1C). One of the clusters (Cluster 5) stood out both in terms of patient survival and cellular composition. The “low-granulocyte” Cluster 5 microenvironment signature was shown to add significant additional prognostic information to standard tumor-specific risk-stratification tools, including the ISS stage; GEP-70 high-risk status; cytogenetic features including amp1q, del17p, adverse translocation t(4;14), and t(14;16); and “double-hit” disease [38] (Fig 1D). Follow-up studies on predisease SMM and MGUS patients revealed that this microenvironment developed as the disease progressed (Fig 1E). Machine-learning techniques identified WBM components that independently impacted survival beyond known high-risk tumor features, with mast cell percentage being the single most predictive factor (Fig 1F). This signature was validated in the STRATUS trial, in which patients with RRMM were significantly enriched for the low-granulocyte phenotype (Fig 1G). The results of this analysis show that the nontumor composition of the MM microenvironment independently impacts survival and can be used to refine risk-stratification approaches.

### What this study adds to existing research

Existing research in MM has tended to focus on tumor mutations and patient clinical characteristics. This is the first large-scale study of the MM TME (i.e., the WBM). It reveals a low-granulocyte microenvironment that is associated with negative patient outcomes and shows how that microenvironment develops during predisease states. This study demonstrates that a WBM granulocyte (i.e., mast cell) signature may be used to stratify patients and identify an elevated risk population that would be missed by existing high-risk criteria.

It is important that this study implicates mast cells and other innate immune cells because most TME research focuses on the adaptive immune system (e.g., T cells). This may be because the deconvolution techniques do a relatively bad job of detecting T cell populations based on our internal validation data set, indicating room for further improvements in deconvolution technology (S1I Fig). The WBM samples in this study contain many fewer mature mast cells than the 4.8% reported in the myeloma literature [27,287]. Although this may be due to differences in staining methods, it emphasizes the need for additional studies of innate immune cells in myeloma patients.

### Strengths and limitations of this study

This study’s great strength is that it has up to 15 years of follow-up data for a high number of MM patients (436) sampled at different time points during treatment (867 samples). The patterns observed in this cohort have been validated in 131 high-risk predisease samples and 295 post-treatment relapse samples in the STRATUS cohort.

This study is limited in that the patients were primarily treated with thalidomide rather than the current front-line drug, lenalidomide (although lenalidomide was used in the STRATUS cohort). This study is also limited by the computational deconvolution used to enumerate cell types. Deconvolution demonstrates poor ability to differentiate cell types in the same lineage (S1D Fig). This challenges the identification of mast cells as the cell type best able to predict patient outcomes. Highly correlated mast cell, eosinophil, neutrophil, and monocyte counts make it difficult to tell whether mast cell estimates reflect another cell type or even a common progenitor. Implications of this limitation are presented in S3 Text.

### Implications and next steps for research

The importance of nonmacrophage myeloid lineage cells in this study implies several plausible partial mechanisms that should be tested experimentally in the context of MM. Mast cells have been shown to play a role in the initiation of antitumor immune response [39], and differential gene-expression analysis supports this observation. Comparison of tumor gene-expression profiles from patients with low mast cell signatures with the other patients yields only 2 genes with significantly lower expression; both are associated with mast cell recruitment and activation (Fig 6B and S7 Fig). Vascular cell adhesion protein 1 is an adhesion protein, utilized by circulating premast cells in order to move into a tissue [40]; thus, tumors lacking this protein likely attract fewer mast cells. Proteoglycan 2 is a mast cell activation protein, the absence of which renders recruited mast cells less likely to be activated and thereby recruited by other immune cells [41].

The mast cell signature is the strongest signal in the data set analyzed, but this does not preclude the role of other related cell types in survival; e.g., low levels of eosinophils are associated with impaired survival and have been previously implicated in the initiation of antitumor immune responses (Fig 6B) [42]. Furthermore, mast cells, eosinophils, neutrophils, and monocytes have linked functions. Mast cells are known to recruit both eosinophils and neutrophils [43]; monocytes and mast cells are often co-recruited [44], and eosinophils are believed to activate mast cells and neutrophils [41]. Thus, it might be profitable for future researchers to purify these cell types (both individually and combined) from MM patient samples. This could lead to a more precise biomarker than the mast cell signature.

Beyond biomarker discovery, in vitro investigations of stromal cells highlighted in this study may be particularly profitable. By illuminating the interactions between these cell types and the tumor, new findings may suggest treatments for myeloma that complement existing therapies. One outstanding question is why patients who enter microenvironment Cluster 5 and leave do not have better outcomes than those who stay in Cluster 5. Has the microenvironment suffered some damage that is undetectable by the assays used here? One way to answer this sort of question would involve reconstructed BM 3D bioassays [45–47]. Models of these kind have shown improved ability to model tumor cell survival in drug-resistant MM. Therefore, they might provide a controlled environment to measure signaling as tumor and microenvironment components interact to determine tumor proliferation and drug resistance.

### Implications and next steps for clinical practice

Mast cell estimates derived from WBM gene expression are a potentially useful biomarker to determine which patients have elevated risk status. As shown in Fig 5 (and S8 Fig), patients with low mast cell estimates (<8.97%) have worse outcomes than patients with higher mast cell estimates, whether or not the patient has been identified as having elevated risk by ISS stage or GEP-70 criteria. Patients who relapse show signs of the same “low-granulocyte” Cluster 5 that develops as MGUS and progresses to MM. If the microenvironment change precedes the malignant cell changes, then it is possibility that the “low-granulocyte” microenvironment may indicate patients who are about to relapse. Taken together, this implies that clinicians should be mindful of the MM WBM tumor microenvironment. WBM gene-expression data can flag elevated risk patients. Enumerations of the cell types present in the WBM of patients with MM, particularly of myeloid lineage cells such as mast cells, eosinophils, neutrophils, and monocytes, could test the conclusions of this study and potentially reveal new ways to treat patients who would otherwise suffer poor outcomes.

## Conclusion

Taken together, the observations in this study provide evidence that the microenvironment is a significant contributor to tumor behavior and treatment response. Further investigation of the microenvironment cellular content may have significant impacts on outcome after immune therapies and, in addition, the role of atypical myeloid cells including granulocytes, myeloid derived suppressor cells, myeloid precursors, and other myeloid lineage cell types should be explored further in this context. To summarize, this article should encourage myeloma clinicians and researchers to study the TME and pay particular attention to granulocytes; their presence (or absence) may contribute to patient survival.

## Supporting information

S1 Text STROBE checklist. The STROBE checklist including indications of where in the text to find the relevant item. STROBE, Strengthening the Reporting of Observational Studies in Epidemiology.
(DOC)

S2 Text Supplementary methods. Additional details about the methods, particularly relevant for replicating the results presented in this publication.
(DOCX)

S3 Text Supplementary discussion. Additional discussion about the limitations of deconvolution and the implications for this publication.
(DOCX)

S1 Table Clinical information for CD138^+^ purified samples. Clinical information for all 436 patients, including file names for the baseline CD138+ samples. CD, cluster of differentiation.
(CSV)

S2 Table Clinical information for WBM samples. Clinical information for all WBM samples, including file names and relevant time point information. WBM, whole bone marrow.
(CSV)

S3 Table Clinical information for MGUS and SMM samples. Clinical information, including file names for the SMM and MGUS samples. MGUS, monoclonal gammopathy of undetermined significance; SMM, smoldering multiple myeloma.
(CSV)

S4 Table Eosinophil IHC quantifications. Eosinophil estimates by IHC and deconvolution, including sample IDs. IHC, immunohistochemistry.
(CSV)

S5 Table Neutrophil IHC quantifications. Neutrophil estimates by IHC and deconvolution, including sample IDs. IHC, immunohistochemistry.
(CSV)

S6 Table Mast and premast IHC quantifications. Mast and premast estimates by IHC and deconvolution, including sample IDs. IHC, immunohistochemistry.
(CSV)

S1 Fig MGSM27 and deconvolution results. (A) Shown is the signature matrix for 27 cell types showing all 601 genes. MGSM27 is publicly available as part of the ADAPTS package (https://cran.r-project.org/web/packages/ADAPTS/index.html). (B) Spillover matrix showing the average deconvolved percentage of cell types (columns) for the purified deconvolved samples (rows). Cells are sorted based on hierarchal clustering based on Pearson correlation coefficient. Also shown are the cell types that were combined to make the 18 cell types used throughout the study. Note that cell types with very small distances were collapsed unless they were biologically very different cell types (e.g., macrophages and dendritic cells). (C) Shown is the comparison between estimated deconvolution and pathologist-determined tumor purity for 423 patients with gene-expression data from purified CD138^+^ samples and flow-based tumor percentages. The left side shows a scatter plot comparing deconvolution and flow-based purity of CD138^+^ samples. Boxes drawn at 75%, 80%, and 90% show that both the deconvolved estimates and clinical estimates indicate high levels of CD138^+^ purity. The right side shows the same data if samples that are more than 90% CD138^+^ are considered “pure.” (D) Comparisons between estimated deconvolution and pathologist-determined tumor percentages for 247 patients with matched pretreatment WBM and CD138^+^ gene-expression data, as well as microscopy and flow-based tumor percentages. (E) Shown is the deconvolved eosinophil estimates and the pathology-estimated percentage for proximal samples taken from 345 patients. Note the pathology estimates show vertical bands consistent with rounding to the nearest half a percent, impeding correlation analysis. A blue X denotes the mean deconvolution percentage of those samples with a pathology estimate of less than 6%, and the dotted blue line shows the regression line for those samples. A green X denotes the mean deconvolution percentage of the remaining samples, and the dotted green line shows their regression line. Importantly, the predictions are in the correct range: 323/345 (94%) of the samples are less than 8% eosinophils as predicted by the deconvolution. (F) Deconvolved neutrophil estimates and the pathology-estimated percentage for proximal samples taken from 356 patients. (G) Deconvolved estimates of PBMC data extracted from E-MTAB-3732: 182 samples. The percentages on the left side show the expected cell types from pathologist-purified peripheral blood mononuclear cells, and those on the right show the deconvolved estimates. The ranges on the right show the mean ± 1 standard deviation. The deconvolved estimates for cell types are calculated by adding the means of all cells of a particular type, as indicated by the color code; e.g., the pink B cell estimates (13%) are the sum of 10% MM.plasma.cells, 2% PlasmaMemory cells, and 1% B.cells.memory. (H) Shows the RMSEs for different deconvolution algorithms to predict the percentage tumor in CD138^+^ purified aspirates and WBM. Some of these samples were replicates that were not included in the main study. (I) shows the Spearman’s correlation coefficient for different algorithms reconstructing the relative order of neutrophils, NK cells, macrophages, and CD3^+^ T cells in a proprietary data set. ADAPTS, Automated Deconvolution Augmentation of Profiles for Tissue Specific cells; CD, cluster of differentiation; DCQ, digital cell quantifier; DSA, digital sorting algorithm; MGSM27, myeloma genome signature matrix 27; MM, multiple myeloma; NK, natural killer; PBMC, peripheral blood mononuclear cell; PCC, Pearson correlation coefficient; RMSE, root mean-square error; WBM, whole bone marrow.
(DOCX)

S2 Fig Additional patient outcomes based on microenvironment cluster. (A) Pretreatment PFS for the “low-granulocyte” Cluster 5 (orange) versus the other 4 clusters. (B) Pretreatment OS for the “low-granulocyte” Cluster 5 (orange) versus the other 4 clusters. (C) Postinduction PFS for the “low-granulocyte” Cluster 5 (orange) versus Clusters 1–4 (blue). (D) Postinduction OS for the “low-granulocyte” Cluster 5 (orange) versus Clusters 1–4 (blue). The *p*-values in the legend compare each cluster to all others with a χ^2^ test. Note that for (A) and (B) the Cluster 5 estimated mean (rmean) and χ^2^ *p*-values are slightly different from that reported in Fig 3. This is due to how having 5 clusters instead of 2 changes the calculation. OS, overall survival; PFS, progression-free survival.
(DOCX)

S3 Fig Characteristics of patients who will relapse. *p*-values from Wilcoxon tests for each cell type, relapse samples versus complete remission samples. Population: 17 patients with a sample within 180 days prior to relapse or 30 days after relapse, excluding baseline samples meeting this criterion; 108 samples taken from patients while in complete remission. Rows in yellow pass an FDR cutoff of 0.20; rows in blue pass an FDR cutoff of 0.05. FDR, false discovery rate; NK, natural killer; N/A, not applicable.
(DOCX)

S4 Fig Kaplan–Meier OS and PFS curves showing how the quartiles of 4 highly correlated cells segment the pretreatment patient population. (A) Eosinophils. (B) Mast cells. (C) Monocytes. (D) Neutrophils. The 2 middle quartiles are combined into a single red line. OS, overall survival; PFS, progression-free survival.
(DOCX)

S5 Fig Gene-set enrichment by bicluster. As an orthogonal approach to validate the biological relevance of low-granulocyte populations in the generation of a high-risk microenvironment, we constructed patient-specific clusters of coexpressed genes (i.e., biclusters) [31] using cMonkey2 separately on WBM and CD138^+^-selected samples. cMonkey2 generates gene clusters that are often highly enriched in genes of related function and/or in related biochemical or regulatory pathways [32–34]. We expected biclusters from this analysis to be enriched for genes driven by specific processes in cell types that are transcriptionally related. By contrasting those biclusters between the 2 data sets, we expected to identify processes that were specific to the high-risk microenvironment. (A) cMonkey2 biclusters significantly correlated with patient PFS. Shown are GO terms enriched in cMonkey2 biclusters (out of a maximum 9,258 clusters for CD138^+^ data and 8,540 clusters for WBM data). The y-axis shows the number of clusters built on the pretreatment WBM samples; the x-axis shows the number of clusters built on CD138^+^ samples. This analysis resulted in 8,540 (potentially redundant) WBM biclusters and 9,258 CD138^+^ biclusters, each containing an average of 30.0 and 30.3 genes, respectively, from 187.0 and 253.6 patients, respectively. After performing functional enrichment analysis using the GO terms [35,36], we found that although many common functions (e.g., cell cycle) were enriched in similar numbers of biclusters between the 2 data sets, many more biclusters generated from the WBM data (approximately 130 biclusters, or approximately 1.5% of all biclusters) were enriched for granulocyte- and myeloid-related genes (specifically, activation or degranulation of neutrophils, leukocytes, and/or myeloid cells) than were biclusters generated from the CD138^+^ data (approximately 15 biclusters, or approximately 0.15%). (B) shows the top GO terms enriched in WBM clusters. The greater number of biclusters enriched for granulocyte-related functions in WBM is a clear indication of the greater expression from cells in the TME in the WBM data set, whereas the small but nonzero number of biclusters from the CD138^+^ data set could be readily explained by the expected 0%–10% CD138^–^ contamination in CD138^+^ samples. In both data sets, >90% of these granulocyte-associated clusters were positively correlated with PFS (i.e., greater expression of these genes was associated with longer PFS, on average; average correlation of 0.092) compared with 48% for the remaining WBM biclusters (average correlation of –0.01; *t* test difference *p* < 10^−16^). Taken together, this biclustering and GO functional discriminatory analysis provides a separate analysis pipeline, which narrows down the relevant cell types present in Cluster 5 to implicate granulocytes as an important correlate of patient outcomes. ATP, adenosine triphosphate; CD, cluster of differentiation; DNA, deoxyribonucleic acid; BP, biological process; ER, endoplasmic reticulum; GO, gene ontology; ncRNA, noncoding ribonucleic acid; PFS, progression-free survival; RNA, ribonucleic acid; SRP, signal-recognition particle; TME, tumor microenvironment; WBM, whole bone marrow.
(DOCX)

S6 Fig IHC. Patient outcomes as determined by IHC for the 290 patients with IHC samples collected at the same time as samples for microarray. Correlations between deconvolved and proximal pathologist-estimated eosinophils and neutrophils (segmented cells) were 0.25; *p* = 1.6 × 10^−5^ and 0.36 and *p* = 2.3 × 10^−10^, respectively. (A) Shown are conditional inference trees and permutation *p*-values using the pathologist-estimated segmented cell percentages to stratify patients by PFS. (B) Shown are conditional inference trees and permutation *p*-values using the pathologist-estimated segmented cell percentages to stratify patients by OS. (C) Shown are the same measurements as (A) using pathologist-estimated eosinophil density. (D) Shown are the same measurements as (B) using pathologist-estimated eosinophil density. (E) shows the comparison between CD117 (c-Kit)-labeled cells for 20 samples in which the tumor was CD117^–^ and deconvolved cell estimates and ratios. The first column shows the correlated coefficient with CD117^+^ cells that morphologically appear to be mature mast cells. The second column shows the *p*-value associated with the mature mast cell correlations. The third column shows the correlated coefficient with CD117^+^ cells that morphologically appear to be immature mast cells. The fourth column shows the *p*-value associated with the immature mast cell correlations. CD, cluster of differentiation; Eos, eosinophils; IHC, immunohistochemistry; NK, natural killer; OS, overall survival; PFS, progression-free survival.
(DOCX)

S7 Fig Top differentially expressed tumor genes as segmented by deconvolved mast cell quartiles. *p*-values were calculated using an FDR-corrected Wilcoxon signed-rank test. DNA, deoxyribonucleic acid; FDR, false discovery rate.
(DOCX)

S8 Fig Fig 5A with combined mast cells and eosinophils. (A) Cox proportional hazards conditional inference tree that selected an optimal combination of attributes from the cell types and clinical characteristics for the 290 pretreatment samples with known cytogenetics and tumor burden estimates. MastEos is the sum of mast cells and eosinophils. (B) Survival curves with statistics for the 4 groups identified in S8A Fig. Eos, eosinophils; GEP-70, 70-gene Prognostic Risk Score; ISS3, International Staging System stage III; mo, months; PFS, progression-free survival.
(DOCX)

S9 Fig Principal component analysis plot of all microarrays that passed quality control by process site and batch. Overlapping ellipses generated by ggbiplot show no separation of data by batch or site.
(DOCX)

S10 Fig Violin plots of multisampled patients across treatment. “1st Samp” shows the distribution for patients who had only 1 sample (black). For patients with multiple samples in a single treatment phase, their sequential time points are colored red (first), light blue (second), dark blue (third), green (fourth), and orange (fifth).
(DOCX)

## Abbreviations

BM: bone marrow
CD: cluster of differentiation
CI: confidence interval
FDR: false discovery rate
GEP-70: 70-gene Prognostic Risk Score
HR: hazard ratio
ISS: International Staging System
MGSM: myeloma genome signature matrix
MGUS: monoclonal gammopathy of undetermined significance
MM: multiple myeloma
NDMM: newly diagnosed multiple myeloma
NK: natural killer
OS: overall survival
PFS: progression-free survival
RMSE: root mean-square error
RRMM: relapsed/refractory multiple myeloma
SMM: smoldering multiple myeloma
STRATUS: Evaluation of Safety of Pomalidomide in Combination With Dexamethasone (Low Dose) in Patients With Refractory or Relapsed and Refractory Multiple Myeloma
TME: tumor microenvironment
TT: Total Therapy
WBM: whole bone marrow

## References

[1] Collins FS, Varmus H. A new initiative on precision medicine. N Engl J Med. 2015;372: 793–5. 10.1056/NEJMp1500523 25635347 PMC5101938

[2] Fonseca R, Abouzaid S, Bonafede M, Cai Q, Parikh K, Cosler L, et al Trends in overall survival and costs of multiple myeloma, 2000–2014. Leukemia. 2017;31: 1915–21. 10.1038/leu.2016.380 28008176 PMC5596206

[3] Attal MA, Lauwers-Cances V, Hulin C, Leleu X, Caillot D, Escoffre M, et al Lenalidomide, bortezomib, and dexamethasone with transplantation for myeloma. N Engl J Med. 2017;376: 1311–20. 10.1056/NEJMoa1611750 28379796 PMC6201242

[4] Stein CK, Pawlyn C, Chavan S, Rasche L, Weinhold N, Corken A, et al The varied distribution and impact of RAS codon and other key DNA alterations across the translocation cyclin D subgroups in multiple myeloma. Oncotarget. 2017;8: 27854–67. 10.18632/oncotarget.15718. 28427158 PMC5438613

[5] Zhou Y, Barlogie B, Shaughnessy JD Jr. The molecular characterization and clinical management of multiple myeloma in the post-genome era. Leukemia. 2009;23: 1941–56. 10.1038/leu.2009.160 19657360 PMC3686133

[6] Broyl A, Hose D, Lokhorst H, de Knegt Y, Peeters J, Jauch A, et al Gene expression profiling for molecular classification of multiple myeloma in newly diagnosed patients. Blood. 2010;116: 2543–53. 10.1182/blood-2009-12-261032 20574050

[7] Kyle RA, Durie BG, Rajkumar SV, Landgren O, Blade J, Merlini G, et al Monoclonal gammopathy of undetermined significance (MGUS) and smoldering (asymptomatic) multiple myeloma: IMWG consensus perspectives risk factors for progression and guidelines for monitoring and management. Leukemia. 2010;24: 1121–7. 10.1038/leu.2010.60 20410922 PMC7020664

[8] Dutta AK, Fink JL, Grady JP, Morgan GJ, Mullighan CG, To LB, et al Subclonal evolution in disease progression from MGUS/SMM to multiple myeloma is characterised by clonal stability. Leukemia. 2019;33: 457–68. 10.1038/s41375-018-0206-x 30046162 PMC6365384

[9] Bianchi G, Munshi NC. Pathogenesis beyond the cancer clone(s) in multiple myeloma. Blood. 2015;125: 3049–58. 10.1182/blood-2014-11-568881 25838343 PMC4432002

[10] Dairaghi DJ, Oyajobi BO, Gupta A, McCluskey B, Miao S, Powers JP, et al CCR1 blockade reduces tumor burden and osteolysis in vivo in a mouse model of myeloma bone disease. Blood. 2012;120: 1449–57. 10.1182/blood-2011-10-384784 22618707 PMC3423783

[11] Iqbal J, Wright G, Wang C, Rosenwald A, Gascoyne RD, Weisenburger DD, et al Gene expression signatures delineate biologic and prognostic subgroups in peripheral T-cell lymphoma. Blood. 2014;123: 2915–23. 10.1182/blood-2013-11-536359 24632715 PMC4014836

[12] Panchabhai S, Kelemen K, Ahmann G, Sebastian S, Mantei J, Fonseca R. Tumor-associated macrophages and extracellular matrix metalloproteinase inducer in prognosis of multiple myeloma. Leukemia. 2016;30: 951–4. 10.1038/leu.2015.191 26202926

[13] Korde N, Kristinsson SY, Landgren O. Monoclonal gammopathy of undetermined significance (MGUS) and smoldering multiple myeloma (SMM): novel biological insights and development of early treatment strategies. Blood. 2011;117: 5573–81. 10.1182/blood-2011-01-270140 21441462 PMC3316455

[14] Nair B, van Rhee F, Shaughnessy JD Jr, Anaissie E, Szymonifka J, Hoering A, et al Superior results of Total Therapy 3 (2003–33) in gene expression profiling-defined low-risk multiple myeloma confirmed in subsequent trial 2006–66 with VRD maintenance. Blood. 2010;115: 4168–73. 10.1182/blood-2009-11-255620 20124509 PMC2879104

[15] Anaissie EJ, van der Rhee F, Hoering A, Waheed S, Alsayed Y, Petty N, et al Comparing toxicities and survival outcomes with Total Therapy 4 (TT4) for 70-gene (R70)-defined low-risk multiple myeloma (MM) to results obtained with Total Therapy 3 protocols TT3A and TT3B. Blood. 2010;116: (suppl 1; abstr 368). 10.1182/blood.V116.21.368.368

[16] Jethava Y, Mitchell A, Zangari M, Waheed S, Schinke C, Thanendrarajan S, et al Dose-dense and less dose-intense Total Therapy 5 for gene expression profiling-defined high-risk multiple myeloma. Blood Cancer J. 2016;6: e453 10.1038/bcj.2016.64 27471869 PMC5030385

[17] Ortiz M, Towfic F, Samur MK, Flynt E, Jang IS, Wang K, et al A high-risk multiple myeloma group identified by integrative multi-omics segmentation of newly diagnosed patients. Blood. 2018;132: (suppl 1; abstr 3165). 10.1182/blood-2018-99-117114

[18] Newman AM, Liu CL, Green MR, Gentles AJ, Feng W, Xu Y, et al Robust enumeration of cell subsets from tissue expression profiles. Nat Methods. 2015;12: 453–7. 10.1038/nmeth.3337 25822800 PMC4739640

[19] Danziger SA, Gibbs DL, Shmulevich I, McConnell M, Trotter MWB, Schmitz F, et al ADAPTS: Automated Deconvolution Augmentation of Profiles for Tissue Specific cells. PLoS ONE. 2019;14: e0224693 10.1371/journal.pone.0224693 31743345 PMC6863530

[20] Altboum Z, Steuerman Y, David E, Barnett-Itzhaki Z, Valadarsky L, Keren-Shaul H, et al Digital cell quantification identifies global immune cell dynamics during influenza infection. Mol Syst Biol. 2014;10: 720 10.1002/msb.134947 24586061 PMC4023392

[21] Torrente A, Lukk M, Xue V, Parkinson H, Rung J, Brazma A. Identification of cancer related genes using a comprehensive map of human gene expression. PLoS ONE. 2016;11: e0157484 10.1371/journal.pone.0157484 27322383 PMC4913919

[22] Hothorn T, Hornik K, Zeileis A. Unbiased recursive partitioning: a conditional inference framework. J Comput Graph Stat. 2006;15: 651–74. 10.1198/106186006X133933

[23] Therneau TM, Grambsch PM. Modeling survival data: extending the Cox model. Berlin: Springer-Verlag; 2000.

[24] Therneau TM, Lumley T. Package “survival”: Survival Analysis [Internet]. 2017 [cited 2018 Aug 20]. https://mran.microsoft.com/snapshot/2017-03-22/web/packages/survival/survival.pdf

[25] Friedman J, Hastie T, Tibshirani R. Regularization paths for generalized linear models via coordinate. J Stat Softw. 2010;33: 1–22. 20808728 PMC2929880

[26] Hothorn T, Zeileis A. partykit: A Modular Toolkit for Recursive Partytioning in R. Journal of Machine Learning Research. 2015;16: 3905–3909. Available from: https://jmlr.org/papers/v16/hothorn15a.html.

[27] Ribatti D, Vacca A, Nico B, Quondamatteo F, Ria R, Minischetti M, et al Bone marrow angiogenesis and mast cell density increase simultaneously with progression of human multiple myeloma. Br J Cancer. 1999;79: 451–5. 10.1038/sj.bjc.6690070 10027312 PMC2362443

[28] Ribatti D, Vacca A, Nico B, Crivellato E, Roncali L, Dammacco F. The role of mast cells in tumour angiogenesis. Br J Haematol. 2001;115: 514–21. 10.1046/j.1365-2141.2001.03202.x 11736931

[29] Simon N, Friedman J, Hastie T, Tibshirani R. Regularization paths for Cox’s proportional hazards model via coordinate descent. J Stat Softw. 2011;39: 1–13. 10.18637/jss.v039.i05 27065756 PMC4824408

[30] Walker BA, Leone PE, Jenner MW, Li C, Gonzalez D, Johnson DC, et al Integration of global SNP-based mapping and expression arrays reveals key regions, mechanisms, and genes important in the pathogenesis of multiple myeloma. Blood. 2006;108: 1733–43. 10.1182/blood-2006-02-005496 16705090

[31] Cheng Y, Church GM. Biclustering of expression data. Proc Int Conf Intell Syst Mol Biol. 2000;8: 93–103. 10977070

[32] Reiss DJ, Plaisier CL, Wu WJ, Baliga NS. cMonkey2: automated, systematic, integrated detection of co-regulated gene modules for any organism. Nucleic Acids Res. 2015;43: e87 10.1093/nar/gkv300 25873626 PMC4513845

[33] Reiss DJ, Baliga NS, Bonneau R. Integrated biclustering of heterogeneous genome-wide datasets for the inference of global regulatory networks. BMC Bioinformatics. 2006;7: 280 10.1186/1471-2105-7-280 16749936 PMC1502140

[34] Danziger SA, Reiss DJ, Ratushny AV, Smith JJ, Plaisier CL, Aitchison JD, et al Bicluster sampled coherence metric (BSCM) provides an accurate environmental context for phenotype predictions. BMC Syst Biol. 2015;9 (Suppl 2): S1 10.1186/1752-0509-9-S2-S1 25881257 PMC4407105

[35] Ashburner M, Ball CA, Blake JA, Botstein D, Butler H, Cherry JM, et al. Gene ontology: tool for the unification of biology. The Gene Ontology Consortium. Nat Genet. 2000;25: 25–9. 10.1038/75556 10802651 PMC3037419

[36] The Gene Ontology Consortium. Expansion of the Gene Ontology knowledgebase and resources. Nucleic Acids Res. 2017;45: D331–8. 10.1093/nar/gkw1108 27899567 PMC5210579

[37] Dimopoulos MA, Palumbo A, Corradini P, Cavo M, Delforge M, Weisel KC, et al. An updated analysis of the STRATUS trial (MM-010): safety and efficacy of pomalidomide plus low-dose dexamethasone (POM + LoDEX) in patients (pts) with relapsed/refractory multiple myeloma (RRMM). Blood. 2015;126: 4225 10.1182/blood.V126.23.4225.4225

[38] Sonneveld P, Avet-Loiseau H, Lonial S, Usmani S, Siegel D, Anderson KC, et al Treatment of multiple myeloma with high-risk cytogenetics: a consensus of the International Myeloma Working Group. Blood. 2016;127: 2955–62. 10.1182/blood-2016-01-631200 27002115 PMC4920674

[39] Oldford SA, Marshall JS. Mast cells as targets for immunotherapy of solid tumors. Mol Immunol. 2015;63: 113–24. 10.1016/j.molimm.2014.02.020 24698842

[40] Abonia JP, Hallgren J, Jones T, Shi T, Xu Y, Koni P, et al. Alpha-4 integrins and VCAM-1, but not MAdCAM-1, are essential for recruitment of mast cell progenitors to the inflamed lung. Blood. 2006;108: 1588–94. 10.1182/blood-2005-12-012781 16670268 PMC1895513

[41] Metcalfe DD, Pawankar R, Ackerman SJ, Akin C, Clayton F, Falcone FH, et al. Biomarkers of the involvement of mast cells, basophils and eosinophils in asthma and allergic diseases. World Allergy Organ J. 2016;9: 7 10.1186/s40413-016-0094-3 26904159 PMC4751725

[42] Carretero R, Sektioglu IM, Garbi N, Salgado OC, Beckhove P, Hämmerling GJ. Eosinophils orchestrate cancer rejection by normalizing tumor vessels and enhancing infiltration of CD8(+) T cells. Nat Immunol. 2015;16: 609–17. 10.1038/ni.3159 25915731

[43] He S, Peng Q, Walls AF. Potent induction of a neutrophil and eosinophil-rich infiltrate in vivo by human mast cell tryptase: selective enhancement of eosinophil recruitment by histamine. J Immunol. 1997;159: 6216–25. 9550425

[44] Trautmann A, Toksoy A, Engelhardt E, Bröcker E-B, Gillitzer R. Mast cell involvement in normal human skin wound healing: expression of monocyte chemoattractant protein-1 is correlated with recruitment of mast cells which synthesize interleukin-4 in vivo. J Pathol. 2000;190: 100–6. 10.1002/(SICI)1096-9896(200001)190:1<100::AID-PATH496>3.0.CO;2-Q 10640999

[45] Reagan MR, Mishima Y, Glavey SV, Zhang Y, Manier S, Lu ZN, et al Investigating osteogenic differentiation in multiple myeloma using a novel 3D bone marrow niche model. Blood. 2014;124(22): 3250–9. https://10.1182/blood-2014-02-558007. 25205118 10.1182/blood-2014-02-558007 PMC4239334

[46] Braham MVJ, Ahlfeld T, Akkineni AR, Minnema MC, Dhert WJA, Öner FC, et al Cellular immunotherapy on primary multiple myeloma expanded in a 3D bone marrow niche model. Oncoimmunology. 2018;7(6): e1434465 https://10.1080/2162402X.2018.1434465. 29872571 10.1080/2162402X.2018.1434465 PMC5980416

[47] Kirshner J, Thulien KJ, Martin LD, Debes Marun C, Reiman T, Belch AR, et al A unique three-dimensional model for evaluating the impact of therapy on multiple myeloma. Blood. 2008;112(7): 2935–45. https://10.1182/blood-2008-02-142430. 18535198 10.1182/blood-2008-02-142430

