# Correction: Bone marrow microenvironments that contribute to patient outcomes in newly diagnosed multiple myeloma: A cohort study of patients in the Total Therapy clinical trials

**DOI:** 10.1371/journal.pmed.1003774

**Published:** 2021-09-08

**Authors:** The PLOS Medicine Staff

There are errors in the author affiliations. The correct affiliations are as follows:

Samuel A. Danziger^1^*, Mark McConnell^1^, Jake Gockley^2^, Mary H. Young^1^, Adam Rosenthal^3^, Frank Schmitz^1^, David J. Reiss^1^, Phil Farmer^4^, Daisy V. Alapat^4^, Amrit Singh^4^, Cody Ashby^4^, Michael Bauer^4^, Yan Ren^1^, Kelsie Smith^1^, Suzana S. Couto^5^, Frits van Rhee^4^, Faith Davies^4^, Maurizio Zangari^4^, Nathan Petty^4^, Robert Z. Orlowski^6^, Madhav V. Dhodapkar^7^, Wilbert B. Copeland^1^, Brian Fox^1^, Antje Hoering^3^, Alison Fitch^1^, Katie Newhall^1^, Bart Barlogie^8^, Matthew W. B. Trotter^9^, Robert M. Hershberg^10^, Brian A. Walker^4^, Andrew P. Dervan^1^, Alexander V. Ratushny^1^‡*, Gareth J. Morgan^4^‡*

**1** Bristol Myers Squibb, Seattle, Washington, United States of America

**2** Sage Bionetworks, Seattle, Washington, United States of America

**3** Cancer Research and Biostatistics, Seattle, Washington, United States of America

**4** Myeloma Center, University of Arkansas for Medical Sciences, Little Rock, Arkansas, United States of America

**5** Genmab, Princeton, New Jersey, United States of America

**6** The University of Texas MD Anderson Cancer Center, Houston, Texas, United States of America

**7** Winship Cancer Institute, Emory University, Atlanta, Georgia, United States of America

**8** Department of Hematology and Medical Oncology, Tisch Cancer Institute, Icahn School of Medicine at Mount Sinai, New York, New York, United States of America

**9** BMS Center for Innovation and Translational Research Europe (CITRE), Seville, Spain

**10** Frazier Life Sciences, Seattle, Washington, United States of America
