# Supplementary material for: Bone marrow microenvironments that contribute to patient outcomes in newly diagnosed multiple myeloma: A cohort study of patients in the Total Therapy clinical trials
Source: PLoS Med. 2020 Nov 4;17(11):e1003323. doi: 10.1371/journal.pmed.1003323 (PMC7641353; doi:10.1371/journal.pmed.1003323)
Supplement: S2 Text — Additional details about the methods, particularly relevant for replicating the results presented in this publication. (DOCX) [file pmed.1003323.s002.docx]

**Bone marrow microenvironments that contribute to patient outcomes in newly diagnosed multiple myeloma: A retrospective study of patients in the Total Therapy clinical trials**

# **S2 Text: Supplementary methods**

Gene-expression data were generated using the Affymetrix Human Genome U133 Plus 2.0 Array (Thermo Fisher Scientific, Waltham, Massachusetts, United States of America) chips from whole bone marrow (WBM) biopsies and CD138^+^-selected myeloma plasma cells. Risk status was assessed by International Staging System stage as defined by the International Myeloma Working Group [1]. CD138^+^ gene-expression data were used to generate the 70-gene Prognostic Risk Score (GEP-70) score [2]. A total of 1,360 Affymetrix Human Genome U133 Plus 2.0 gene-expression microarrays were derived from WBM biopsies from 461 patients. Samples were assessed for quality control using the *arrayQualityMetrics* R Bioconductor package using default parameters to determine exclusion criteria [3]. Samples were excluded from analysis if either: (1) clustering distance was abnormally large, array probe distribution was significantly different from the population; (2) standard deviation of the probe intensity distribution was significantly different from the total cohort; or (3) the relative log expression or normal unscaled standard error was significantly differently distributed from the population. Samples that passed quality control comprised pre-treatment samples (n = 363), post-treatment (n = 331), post-transplantation (n = 95), post-consolidation (n = 57), and post-maintenance (n = 237). Sample retention was similar across sampling point (baseline, 76.1%; post-treatment, 77.5%; post-transplantation, 74.8%; post-consolidation, 82.6%; and post-maintenance, 82.0%) indicating that filtering metrics did not appear to operate preferentially to sampling point. Batch correction was performed for both the site from which the microarrays were run as well as a previously identified change in processing reagents utilizing ComBat (Bioconductor) [4]. The principal component analysis revealed no readily identifiable batch substructure (S9 Fig).

Samples were filtered to produce an analysis series with no more than a single sample per patient at a given time point (S10 Fig). In the event that a patient was sampled multiple times before treatment, the most recent sample was chosen as this reflected the proper course of treatment relative to those patients with matched post-treatment size. Final filtering involved removing samples that did not appear to feasibly follow the course of treatment. This final analysis cohort contained 436 patients and 867 samples, which were distributed as follows: baseline (n = 354), post-treatment (n = 245), post-transplantation (n = 83), post-consolidation (n = 51), post-maintenance (n = 134). Among patients with a sample before treatment, 189 had a post-treatment sample, 63 had a post-transplantation sample, 39 had a post-consolidation sample, and 104 had a post-maintenance sample. The total number of samples in this sub-cohort was 749.

**Additional validation of deconvolution accuracy**

We also showed that the algorithm could accurately estimate the identity of other (BM) bone marrow cellular population (S1B Fig). For 24 of 27 cell types, the labeled cell type was the highest percentage component of the mix. For the remaining three cell types, the samples deconvolved as a mix of cell types with similar lineages to the labeled cell type. Osteoclasts deconvolved as 22% M2 macrophages, 20% M0 macrophages, and 19% osteoclasts. Eosinophils deconvolved as 17% CD138^+^-purified cells, 16% monocytes, 15% M2 macrophages, and 11% eosinophils. Myeloma plasma cells obtained from public data [5] deconvolved as 54% CD138^+^-purified cells, 22% plasma cells, 13% memory plasma cells, and 9% MM plasma cells. All cell types showed spillover to similar lineage cells, e.g. activated mast cells deconvolved as 28% activated mast cells, 25% resting mast cells, and the remainder was a mix of other granulocytes and progenitor cells. Consequently, we combined the counts for highly related cell types to arrive at 19 distinct cell types that could be differentiated.

**Myeloma Genome Signature Matrix 27 (MGSM27) development and pseudo CD138^−^ gene-expression construction**

To develop an approach applicable to the BM of plasma cell disorders, we expanded the LM22 signature matrix for 22 leukocyte cell types [6] into MGSM27 by adding gene-expression profiles for five additional BM-specific cell types [7], including adipocytes [8], malignant plasma cells [8], osteoclasts [8], plasma memory cells [6], and osteoblasts (E-MTAB-4152) [7]. To differentiate these additional cell types, we included 54 additional genes.

To eliminate the contribution of malignant cells to gene-expression data, the total plasma cells were removed and the remaining cell types were rescaled to 100%. To estimate the gene-expression levels from each cell type, we considered only those 6,432 genes for which we had expression levels for nearly all the 27 cell types. We then calculated the non-tumor portion of the expression by subtracting expression in the templates in the tumor type weighted by the deconvolved tumor expression**.** Once the expression levels for the 6,432 genes were calculated for all patients, the gene-expression levels for each patient were rescaled so that all patients had the same maximum gene expression.

For example, if Patient 1 had a log_2_ expression level of 10 for Gene 1 and the four tumor cell types each had a log_2_ expression of five, adding up to a total expression level of 20, with the log_2_ expression for the remaining 23 cell types adding up to 80. If deconvolution predicted that Patient 1 had 60% tumor, then the non-tumor portion of the total expression would be 0.4 × 80/(0.6 × 20 + 0.4 × 80) = 0.73. Therefore, the log_2_ expression of the non-tumor portion of Gene 1 in Patient 1 was 0.73 × 10, or 7.3.

Rescaling gene-expression levels like this introduces unavoidable artefacts related to dynamic range. If a sample is 90% tumor, the remaining 10% will be split between all stromal components. If that 10% is then amplified to be 100%, the results will show reduced granularity.

**Gene-expression clustering**

Unsupervised clustering of the top 10% most variable estimated non-tumor gene-expression profiles (643 genes) across all time points was performed using the R package *ConsensusClusterPlus* (Bioconductor) [9]. Bayesian information criterion (BIC) was used to select the optimal number of clusters [5]. The cluster centroids were calculated based on the median expression of each of the genes for the patient samples belonging to that cluster, and the sum of the squared distances was taken for the entire patient cluster. The residual sum squared distances across all clusters were used to calculate the BIC score, with the assumption of a Gaussian special case [10], as follows:

BIC = *n* × ln(RSS/*n*) + ln(*n*) × *k*, where *n* is the number of observations, RSS is the residual sum of squares, and *k* is the number of clusters.

The maximum possible number of clusters was set to the square root of the number of samples; thus, for the 867 patient samples, the optimal number of clusters determined by the algorithm was 13 out of a possible maximum of 29 clusters. Several of these microenvironment clusters comprised very few patient samples and therefore were combined into an orphan cluster. Eight clusters, each containing less than 5% of the total samples, were assigned to the orphan cluster. The remaining five larger clusters are shown in Fig 2.

**Survival analysis statistics**

For supervised machine-learning survival analysis, cell-type estimates for cells with similar functions and gene-expression signatures were added together to create 19 cell types for assessment (S1F Fig). To determine which combinations of cell types and cytogenetic characteristics best separated patients with relative long and short overall survival and progression-free survival, we used conditional inference trees [11] with the Cox proportional hazards model [12], which conveniently included permutation tests to help determine generalizability.

Kaplan–Meier curves were generated using the R Survival package (Bioconductor) [13] and *p*-values were estimated with a Chi^2^ test. Regression analysis (including lasso regression) to evaluate feature performance was performed using Cox regression in the R package *GLMnet* (Bioconductor) [13,14]. Decision trees were generated using the conditional inference trees algorithm, which accounts for censoring using a Cox proportional hazards model and only adds variables when they pass a *p*-value cutoff as calculated by a permutation test [11,15].

**Gene expression deconvolution and machine-learning approaches**

To construct an MGSM27, we performed a t-test comparing each of the five new cell types to all other cell types and removed any gene that did not pass a false discovery rate of 0.3. Genes were then ranked according to expression change and genes appearing in the top 100 for any new cell type were added to the cell signature matrix. The missForest package [16] was used to impute the expression level of any missing genes. We determined the optimal number of genes to retain by calculating the condition numbers [17] for additional genes, smoothing the curve using Tukey's (running median) smoothing [18] to find stable inflection points and selecting the minimum condition number. On the basis of this, 54 additional genes were added, increasing the genes in LM22 from 547 to 601. MGSM27 was then quartile normalized to increase the contrast between different cell types.

After DCQ deconvolution, the coefficients reported were scaled so that the sum of all coefficients added up to 100% with any negative coefficients assigned to an “other” cell type. The computational deconvolution has several important limitations, including that it would only look for cell types that are in the deconvolution matrix. Other cell types (e.g. hemopoietic cells are almost certainly in BM but not in the deconvolution matrix) will be usually assigned to some cell type besides “other”. Therefore, when the deconvolution algorithm reports 12.5% eosinophils, it is likely that some percentage of these cells are actually cell types that are in the sample but not in the deconvolution matrix. Therefore, the real percentage of eosinophils may be more like 4% or 8%.

Furthermore, a deconvolution algorithm cannot differentiate between two low-expression cells and one cell with twice as much gene expression. This may be particularly relevant for neutrophils, which are less transcriptionally active than other cell types [19]. Therefore, we would expect neutrophils and similar cell types to be underestimated and by extension all other cell types overestimated.

**Gene expression biclustering using cMonkey2**

Genes were combined into condition-specific biclusters [20] using the cMonkey2 algorithm [1,21–23]. We performed 10 repeated cMonkey2 runs on each data set to find 10,800 biclusters filtered to 9,258 for the CD138^+^ data and 9,000 biclusters filtered to 8,540 for the pre-treatment WBM samples. We then performed functional gene set enrichment to identify biclusters significantly enriched for genes of a single Gene Ontology (GO) [24,25] biological process (BP) and counted identified GO BPs that were significantly enriched in multiple biclusters (this represents a repeated observation and implies that the detection is more reliable). To determine the relationship between these biclusters and patient outcomes, we compared the number of biclusters with each GO BP term against their average Pearson correlation with progression-free survival. This revealed 107 genes that occur in > 10 of those granulocyte-enriched WBM biclusters.

**Cell types determined by immunohistochemistry and flow cytometry**

Plasma cells were counted using conventional bright-field microscopy and multiparameter flow cytometry [26]. Approximate estimation of BM cells was performed using May–Grünwald–Giemsa staining of BM aspirate and a 200-cell differential count under × 1,000 magnification. BM aspirate composed of populations of plasma cells, lymphocytes, monocytes, and myeloid cells that included all stages of granulocytes, eosinophils, basophils, mast cells, and erythroid cells. Flow cytometry estimates of tumor proportion reflect a CD38^hi^/CD138^hi^ enumeration divided by the total number of nucleated cells.

**References**

1. Sonneveld P, Avet-Loiseau H, Lonial S, Usmani S, Siegel D, Anderson KC, et al. Treatment of multiple myeloma with high-risk cytogenetics: a consensus of the International Myeloma Working Group. Blood. 2016; 127:2955–62. https://doi.org/10.1182/blood-2016-01-631200 PMID: 27002115
2. Weinhold N, Heuck CJ, Rosenthal A, Thanendrarajan S, Stein CK, Van Rhee F, et al*.* The clinical value of molecular subtyping multiple myeloma using gene expression profiling. Leukemia. 2016; 30:423–30. https://doi.org/10.1038/leu.2015.309 PMID: 26526987
3. Kauffmann A, Gentleman R, Huber W. arrayQualityMetrics—a bioconductor package for quality assessment of microarray data. Bioinformatics. 2009; 25:415–6. https://doi.org/10.1093/bioinformatics/btn647 PMID: 19106121
4. Stein CK, Qu P, Epstein J, Buros A, Rosenthal A, Crowley J, et al. Removing batch effects from purified plasma cell gene expression microarrays with modified ComBat. BMC Bioinformatics. 2015; 16:63. https://doi.org/10.1186/s12859-015-0478-3 PMID: 25887219
5. Schwarz G. Estimating the dimension of a model. Ann Stat. 1978; 6:461–4.
6. Mahévas M, Patin P, Huetz F, Descatoire M, Cagnard N, Bole-Feysot C, et al. B cell depletion in immune thrombocytopenia reveals splenic long-lived plasma cells. J Clin Invest. 2013; 123:432–42. https://doi.org/10.1172/JCI65689 PMID: 23241960
7. Kolesnikov N, Hastings E, Keays M, Melnichuk O, Tang YA, Williams E, et al. ArrayExpress update--simplifying data submissions. Nucleic Acids Res. 2015;43:D1113-6. https://doi.org/10.1093/nar/gku1057 PMID: 25361974
8. Newman AM, Liu CL, Green MR, Gentles AJ, Feng W, Xu Y, et al*.* Robust enumeration of cell subsets from tissue expression profiles. Nat Methods. 2015; 12:453–7. https://doi.org/10.1038/nmeth.3337 PMID: 25822800
9. Wilkerson MD, Hayes DN. ConsensusClusterPlus: a class discovery tool with confidence assessments and item tracking. Bioinformatics. 2010; 26:1572–3. https://doi.org/10.1093/bioinformatics/btq170 PMID: 20427518
10. Priestley MB. Spectral analysis and time series. Cambridge, MA: Academic Press; 1982.
11. Hothorn T, Hornik K, Zeileis A. Unbiased recursive partitioning: a conditional inference framework. J Comput Graph Stat*.* 2006; 15:651–74. https://doi.org/10.1198/106186006X133933.
12. Therneau TM, Grambsch PM. Modeling survival data: extending the Cox model. Berlin: Springer-Verlag; 2000.
13. Therneau TM, Lumley T. (original S->R port and maintainer until 2009), survival: survival analysis. 2017. Available from: https://mran.microsoft.com/snapshot/2017-03-22/web/packages/survival/survival.pdf
14. Friedman J, Hastie T, Tibshirani R. Regularization paths for generalized linear models via coordinate. J Stat Softw. 2010; 33:1–22. PMID: 20808728
15. Hothorn T, Hornik K, Strobl C, Zeileis A. party: a laboratory for recursive partitioning. 2018. Available from: https://cran.rproject.org/web/packages/party/vignettes/party.pdf
16. Stekhoven DJ, Bühlmann P. MissForest—non-parametric missing value imputation for mixed-type data. Bioinformatics. 2012; 28:112–8. https://doi.org/10.1093/bioinformatics/btr597
17. Belsley DA, Kuh E, Welsch RE. Regression diagnostics: identifying influential data and sources of collinearity. Hoboken, NJ: John Wiley; 2005.
18. Tukey JW. Exploratory data analysis. London: Pearson; 1977.
19. Louis NA, Parkos, CA. The neutrophil. In: Mestecky J, Strober W, Russel MW, Kelsall BL, Cheroutre H, Lambrecht BN, editors. Mucosal immunology. 4th ed. Cambridge, MA: Academic Press; 2015. pp 915–29.
20. Cheng Y, Church GM. Biclustering of expression data. Proc Int Conf Intell Syst Mol Biol. 2000; 8:93–103. PMID: 10977070
21. Reiss DJ, Plaisier CL, Wu W-J, Baliga NS. cMonkey2: automated, systematic, integrated detection of co-regulated gene modules for any organism. Nucleic Acids Res. 2015; 43:e87. https://doi.org/10.1093/nar/gkv300 PMID: 25873626
22. Reiss DJ, Baliga N, Bonneau R. Integrated biclustering of heterogeneous genome-wide datasets for the inference of global regulatory networks. BMC Bioinformatics. 2006; 7:280. https://doi.org/10.1186/1471-2105-7-280 PMID: 16749936
23. Danziger SA, Reiss DJ, Ratushny AV, Smith JJ, Plaisier CL, Aitchison JD, et al. Bicluster sampled coherence metric (BSCM) provides an accurate environmental context for phenotype predictions. BMC Syst Biol. 2015; 9:(suppl 2; S1). https://doi.org/10.1186/1752-0509-9-S2-S1 PMID: 25881257
24. Ashburner M, Ball CA, Blake JA, Botstein D, Butler H, Cherry JM, et al*.* Gene ontology: tool for the unification of biology. The Gene Ontology Consortium. Nat Genet. 2000; 25:25–9. https://doi.org/10.1038/75556 PMID: 10802651
25. The Gene Ontology Consortium. Expansion of the Gene Ontology knowledgebase and resources. Nucleic Acids Res. 2017; 45:D331–8. https://doi.org/10.1093/nar/gkw1108 PMID: 27899567
26. Paiva B, Vidriales MB, Pérez JJ, Mateo G, Montalbán MA, Mateos MV, et al. Multiparameter flow cytometry quantification of bone marrow plasma cells at diagnosis provides more prognostic information than morphological assessment in myeloma patients. Haematologica. 2009; 94:1599–1602. https://doi.org/10.3324/haematol.2009.009100 PMID: 19880781
