# Supplementary material for: Bone marrow microenvironments that contribute to patient outcomes in newly diagnosed multiple myeloma: A cohort study of patients in the Total Therapy clinical trials
Source: PLoS Med. 2020 Nov 4;17(11):e1003323. doi: 10.1371/journal.pmed.1003323 (PMC7641353; doi:10.1371/journal.pmed.1003323)
Supplement: S3 Text — Additional discussion about the limitations of deconvolution and the implications for this publication. (DOCX) [file pmed.1003323.s003.docx]

**Bone marrow microenvironments that contribute to patient outcomes in newly diagnosed multiple myeloma: A cohort study of patients in the Total Therapy clinical trials**

**S3 Text: Supplementary discussion**

**Implications of potential artefacts of the deconvolution algorithm.**

Deconvolution demonstrates poor ability to differentiate related cell types, especially those in the same lineage (e.g. osteoclasts and macrophages in S1D Fig). Thus, granulocyte estimates (e.g. of mast cells) may be interpreted as representing both mature and immature myeloid cells, thereby providing a proxy for dysregulation of myeloid differentiation. Attempts to validate mast cell estimates using CD117 (also called c-Kit) in 20 frozen samples of CD117^−^ tumor cells revealed very small numbers of morphologically mature CD117^+^ mast cells (much lower than the previously reported 4.8% of cells in active myeloma) [1,2] and these CD117^+^ cells did not correlate with mast cells estimates obtained by deconvolution (S6E Fig). However, morphologically immature CD117^+^ mast cell precursor-like cells correlated with several of the cell types depleted in Cluster 5 (Fig 3D) including mast cells. This finding suggests potential for tumor-mediated alteration of the myeloid differentiation process, leading to fewer myeloid lineage cells except those that deconvolve as apparently ineffective M1 macrophages [3] and dendritic cells [4]. These cells may actually be misidentified monocyte myeloid-derived suppressor cells (M-MDSCs). Like macrophages and dendritic cells, M-MDSCs are derived from monocytes. However, they are known to impede immune response by suppressing effector T cell function [5–7]. This might explain why Cluster 5 has elevated CD8^+^ T cell estimates as CD8^+^ T cells present in tumors are often ineffective owing to exhaustion, dysfunction, [8] or MDSCs [5–7] despite the more common association with a powerful immune response.

This latter hypothesis regarding tumor-mediated modification of the tumor microenvironment can be supported by genes identified as differentially expressed by tumor cells associated with reduced mast cell levels (S7 Fig). Proteoglycan 2 is a major component of the eosinophil granules and impacts tumor necrosis factor-α and interleukin-8, suggesting a role in modifying tumor microenvironment immune content that could be central to establishing the “low granulocyte” microenvironment Cluster 5. Studies of the extracellular matrix in monoclonal gammopathy of undetermined significance and multiple myeloma (MM) have previously associated proteoglycan 2 and secreted proteins encoded by interferon signaling and response genes with a role in MM pathogenesis that may render the microenvironment more favorable to the tumor [9,10–18]. In addition, vascular cell adhesion protein 1 (VCAM1) and family with sequence similarity 133 member A (FAM133A; a cancer testis antigen) are relevant membrane proteins in a subset of MM that may affect cells in the microenvironment that bind around the tumor [19,20].

Other genes that appear directly related to tumor growth, e.g. changes in cell cycle regulation and apolipoprotein B MRNA editing enzyme catalytic subunit 3B (APOBEC3B) expression, may be less relevant. Similarly, hepatocyte growth factor and its receptor c-Met, expressed on clonal plasma cells in approximately 50% of clinical MM cases, suggest an autocrine loop may be established in this high-risk state that enhances signaling via the MEK/ERK pathway.

The digital cell quantifier (DCQ) deconvolution algorithm is also susceptible to artefacts caused by elastic net regression: the shrinkage may cause the coefficients for some low abundance cell types to be shrunk to zero. This may cause some cell types to be estimated incorrectly as zero percent of the sample, causing the average for several samples to be lower than it otherwise might be. For example, S1G Fig shows an underestimation of the T cell population in peripheral blood mononuclear cells. Therefore, the reported CD8^+^ T cell enrichment in Cluster 5 may be an artefact caused by many patients predicted to have zero T cells.

**References**

1. Ribatti D, Vacca A, Nico B, Quondamatteo F, Ria R, Minischetti M, et al. Bone marrow angiogenesis and mast cell density increase simultaneously with progression of human multiple myeloma. Br J Cancer. 1999; 79:451–5. https://doi.org/10.1038/sj.bjc.6690070 PMID: 10027312
2. Ribatti D, Vacca A, Nico B, Crivellato E, Roncali L, Dammacco F. The role of mast cells in tumour angiogenesis. Br J Haematol. 2001; 115:514–21. https://doi.org/10.1046/j.1365-2141.2001.03202.x PMID: 11736931
3. Cavnar MJ, Zeng S, Kim TS, Sorenson EC, Ocuin LM, Balachandran VP, et al. KIT oncogene inhibition drives intratumoral macrophage M2 polarization. J Exp Med. 2013; 210:2873–86. https://doi.org/10.1084/jem.20130875. PMID: 24323358
4. Oriss TB, Krishnamoorthy N, Ray P, Ray A. Dendritic cell c-kit signaling and adaptive immunity: implications for the upper airways. Curr Opin Allergy Clin Immunol. 2014; 14:7–12. https://doi.org/0.1097/ACI.0000000000000019. PMID: 24300419
5. Görgün G, Samur MK, Cowens KB, Paula S, Bianchi G, Anderson JE, et al. Lenalidomide enhances immune checkpoint blockade induced immune response in multiple myeloma. Clin Cancer Res. 2015; 21:4607–18. https://doi.org/10.1158/1078-0432.CCR-15-0200 PMID: 25979485
6. Bronte V, Brandau S, Chen SH, Colombo MP, Frey AB, Greten TF, et al. Recommendations for myeloid-derived suppressor cell nomenclature and characterization standards. Nat Commun. 2016; 7:12150. https://doi.org/10.1038/ncomms12150 PMID: 27381735
7. Calvert RD, Fleet JC, Chen Y, Pothen A, Rajwa B, Fournier PG, et al. Monocytic myeloid derived suppressor cells (M-MDSC) from spleen are multipotent while tumor M-MDSC have limited plasticity. Cancer Res. 2018; 78:(suppl; abstr 4741). https://doi.org/10.1158/1538-7445.AM2018-4741
8. Li H, van der Leun AM, Yofe I, Lubling Y, Gelbard-Solodkin D, van Akkooi ACJ, et al. Dysfunctional CD8 T cells form a proliferative, dynamically regulated compartment within human melanoma. Cell. 2019; 176:775–89. https://doi.org/10.1016/j.cell.2018.11.043. PMID: 30595452
9. Broyl A, Hose D, Lokhorst H, de Knegt Y, Peeters J, Jauch A, et al. Gene expression profiling for molecular classification of multiple myeloma in newly diagnosed patients. Blood. 2010; 116:2543–53. https://doi.org/10.1182/blood-2009-12-261032 PMID: 20574050
10. Zhan F, Hardin J, Kordsmeier B, Bumm K, Zheng M, Tian E, Sanderson R, et al. Global gene expression profiling of multiple myeloma, monoclonal gammopathy of undetermined significance, and normal bone marrow plasma cells. Blood. 2002; 99:1745–57. https://doi.org/10.1182/blood.v99.5.1745 PMID: 11861292
11. Rampa C, Tian E, Våtsveen TK, Buene G, Slørdahl TS, Børset M, et al. Identification of the source of elevated hepatocyte growth factor levels in multiple myeloma patients. Biomark Res. 2014; 2:8. https://doi.org/10.1186/2050-7771-2-8 PMID: 24716444
12. Kawano Y, Zavidij O, Park J, Moschetta M, Kokubun K, Mouhieddine TH, et al. Blocking IFNAR1 inhibits multiple myeloma–driven Treg expansion and immunosuppression. J Clin Invest. 2018; 128:2487–99. https://doi.org/10.1172/JCI88169 PMID: 29558366
13. Glavey SV, Naba A, Manier S, Clauser K, Tahri S, Park J, et al. Proteomic characterization of human multiple myeloma bone marrow extracellular matrix. Leukemia. 2017; 31:2426–34. https://doi.org/10.1038/leu.2017.102 PMID: 28344315
14. Derksen PW, de Gorter DJ, Meijer HP, Bende RJ, van Dijk M, Lokhorst HM, et al. The hepatocyte growth factor/Met pathway controls proliferation and apoptosis in multiple myeloma. Leukemia. 2003; 17:764-74. https://doi.org/10.1038/sj.leu.2402875 PMID: 12682635
15. Feng B, Ding B, Sun Y, Guo N. Gene expression profiling based on microarray among monoclonal gammopathy of undetermined significance, smoldering multiple myeloma and multiple myeloma. Int J Clin Exp Med. 2017; 10:767–73.
16. Hu Y, Song W, Cirstea D, Lu D, Munshi NC, Anderson KC. CSNK1α1 mediates malignant plasma cell survival. Leukemia. 2015; 29:474–82. https://doi.org/10.1038/leu.2014.202 PMID: 24962017
17. Mao H, Wang M, Cao B, Zhou H, Zhang Z, Mao X. Interferon-stimulated gene 15 induces cancer cell death by suppressing the NF-κB signaling pathway. Oncotarget. 2016; 7:70143–151. https://doi.org/10.18632/oncotarget.12160 PMID: 27659523
18. Fensterl V, Sen GC. The ISG56/IFIT1 gene family. J Interferon Cytokine Res. 2011; 31:71–8. https://doi.org/10.1089/jir.2010.0101 PMID: 20950130
19. van Duin M, Broyl A, de Knegt Y, Goldschmidt H, Richardson PG, Hop WC, et al. Cancer testis antigens in newly diagnosed and relapse multiple myeloma: prognostic markers and potential targets for immunotherapy. Haematologica. 2011; 96:1662–9. https://doi.org/10.3324/haematol.2010.037978 PMID: 21791470
20. Michigami T, Shimizu N, Williams PJ, Niewolna M, Dallas SL, Mundy GR, et al. Cell-cell contact between marrow stromal cells and myeloma cells via VCAM-1 and α4β1-integrin enhances production of osteoclast-stimulating activity. Blood. 2000; 96:1953–60. PMID: 10961900
