# Supplementary material for: Bone marrow microenvironments that contribute to patient outcomes in newly diagnosed multiple myeloma: A cohort study of patients in the Total Therapy clinical trials
Source: PLoS Med. 2020 Nov 4;17(11):e1003323. doi: 10.1371/journal.pmed.1003323 (PMC7641353; doi:10.1371/journal.pmed.1003323)
Supplement: S1 Fig — (A) Shown is the signature matrix for 27 cell types showing all 601 genes. MGSM27 is publicly available as part of the ADAPTS package (https://cran.r-project.org/web/packages/ADAPTS/index.html). (B) Spillover matrix showing the average deconvolved percentage of cell types (columns) for the purified deconvolved samples (rows). Cells are sorted based on hierarchal clustering based on Pearson correlation coefficient. Also shown are the cell types that were combined to make the 18 cell types used throughout the study. Note that cell types with very small distances were collapsed unless they were biologically very different cell types (e.g., macrophages and dendritic cells). (C) Shown is the comparison between estimated deconvolution and pathologist-determined tumor purity for 423 patients with gene-expression data from purified CD138+ samples and flow-based tumor percentages. The left side shows a scatter plot comparing deconvolution and flow-based purity of CD138+ samples. Boxes drawn at 75%, 80%, and 90% show that both the deconvolved estimates and clinical estimates indicate high levels of CD138+ purity. The right side shows the same data if samples that are more than 90% CD138+ are considered “pure.” (D) Comparisons between estimated deconvolution and pathologist-determined tumor percentages for 247 patients with matched pretreatment WBM and CD138+ gene-expression data, as well as microscopy and flow-based tumor percentages. (E) Shown is the deconvolved eosinophil estimates and the pathology-estimated percentage for proximal samples taken from 345 patients. Note the pathology estimates show vertical bands consistent with rounding to the nearest half a percent, impeding correlation analysis. A blue X denotes the mean deconvolution percentage of those samples with a pathology estimate of less than 6%, and the dotted blue line shows the regression line for those samples. A green X denotes the mean deconvolution percentage of the remaining samples, and the dotted green [file pmed.1003323.s010.docx]

**Bone marrow microenvironments that contribute to patient outcomes in newly diagnosed multiple myeloma: A retrospective study of patients in the Total Therapy clinical trials**

**Supporting information**

**S1 Fig. MGSM27 and deconvolution results**


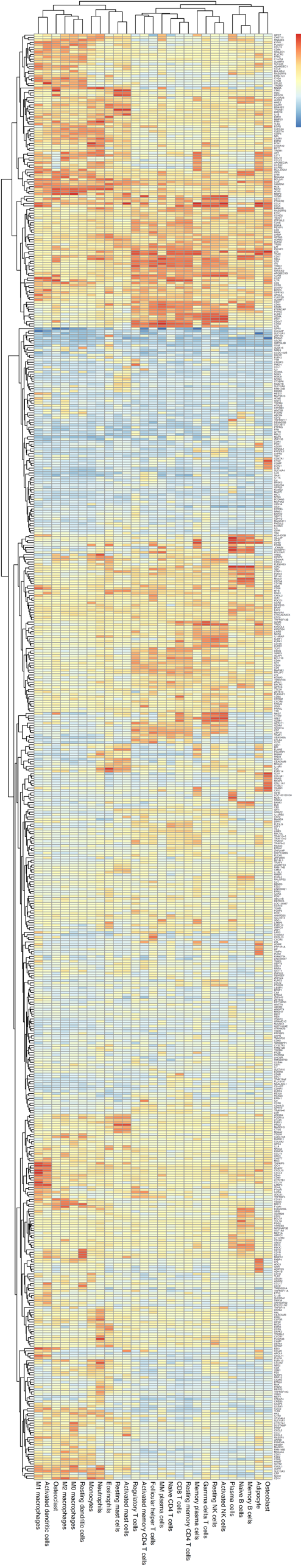
**A**


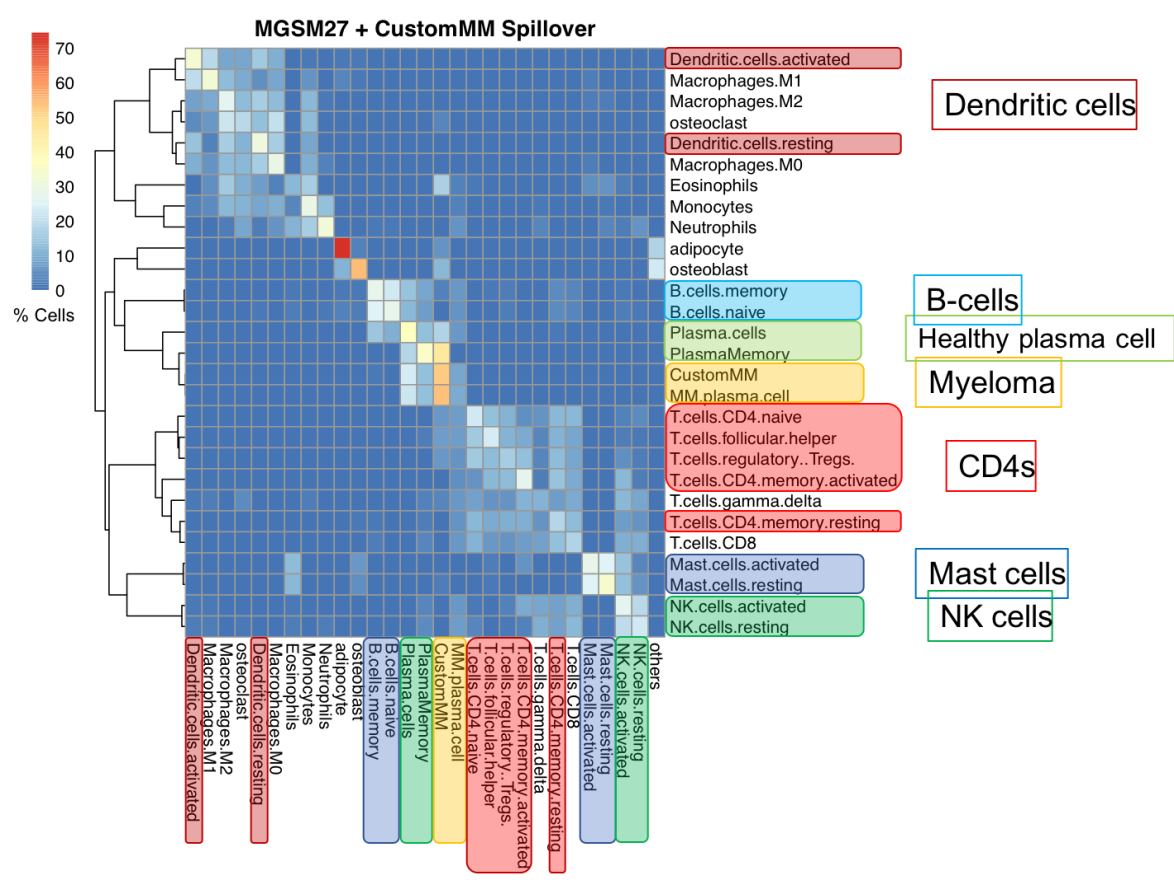
**B**

**C**

**Estimated deconvolution and pathologist-determined tumor purity for 423 patients**

Correlation = 0.31; *p* = 6.1 × 10^−11^; RMSE = 9.37%

75% purity = 92%; 80% purity = 88%; 90% purity = 70%

**CD138 post sort tumor percentage (%)**

**Deconvolved tumor (%)**


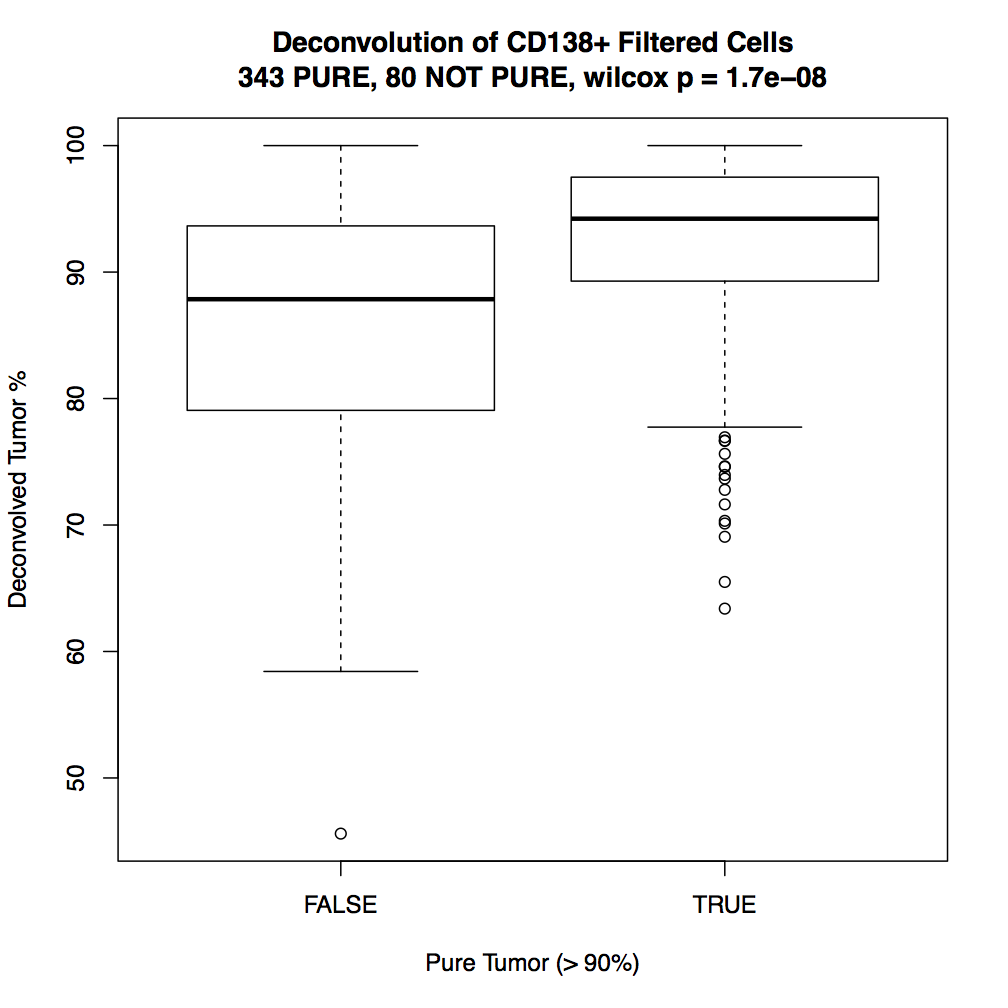


**Deconvolution of CD138^+^ filtered cells**

343 pure, 80 not pure, *p* = 1.7 × 10^−8^

**Deconvolved tumor percentage (%)**

**Pure tumor (> 90%)**

**D**

**D**


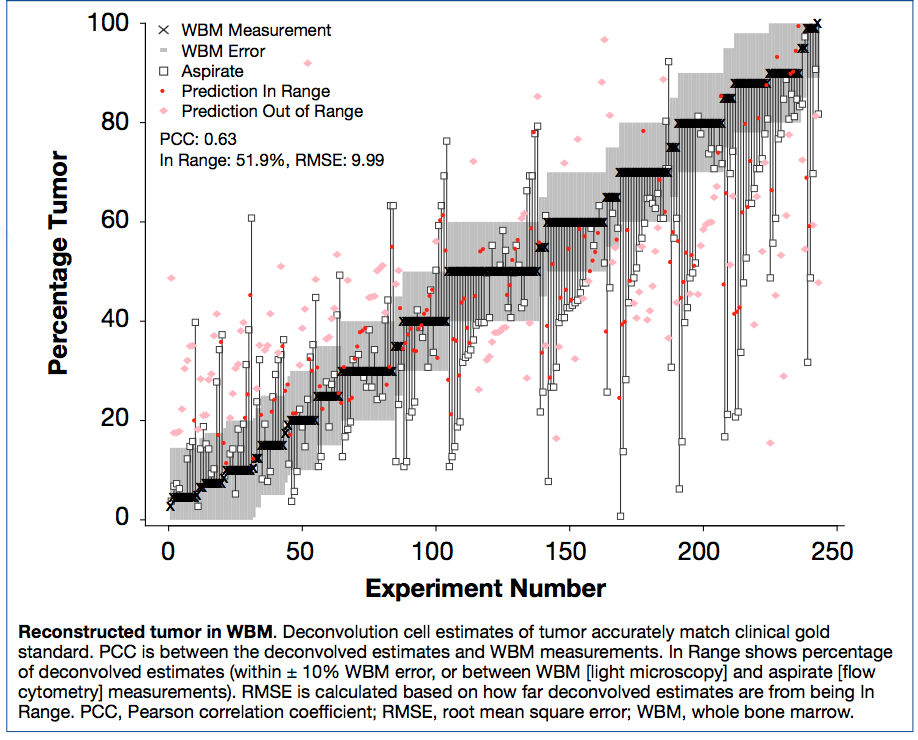


**Tumor percentage (%)**

**Experiment number**

**Tumor percentage (%)**

**Experiment number**

**E**

**Deconvolution percentage (%)**

**Deconvolved eosinophil estimates and the pathology-estimated percentage**

Rho = 0.27; *p* = 5.2 × 10^−7^; RMSE = 3.047; N = 345

**Pathology-estimated percentage (%)**

**F**


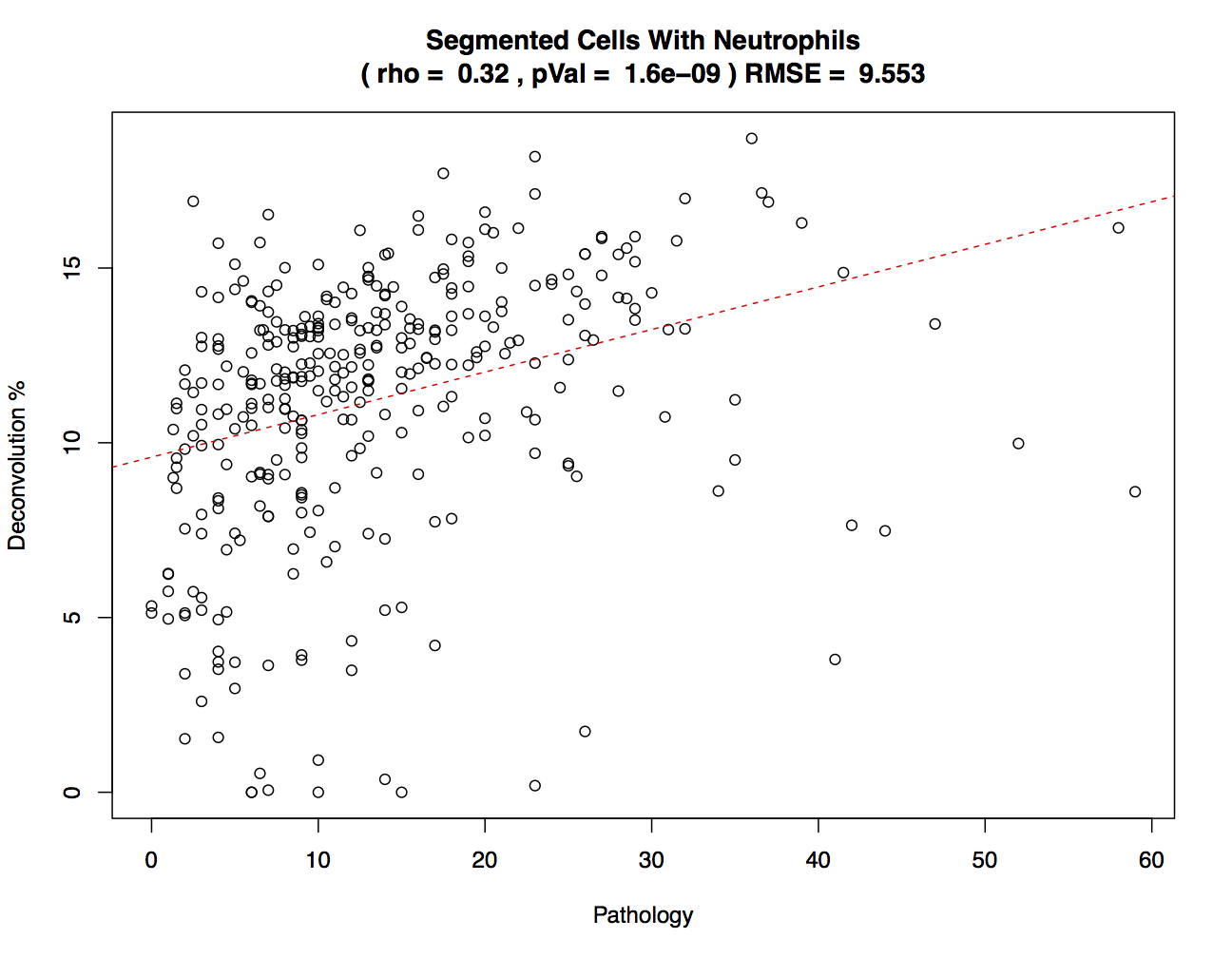


**Deconvolved neutrophil estimates and the pathology-estimated percentage**

Rho = 0.32; *p* = 1.6 × 10^−9^; RMSE = 9.553; N = 356

**Deconvolution percentage (%)**

**Pathology-estimated percentage (%)**

**G**


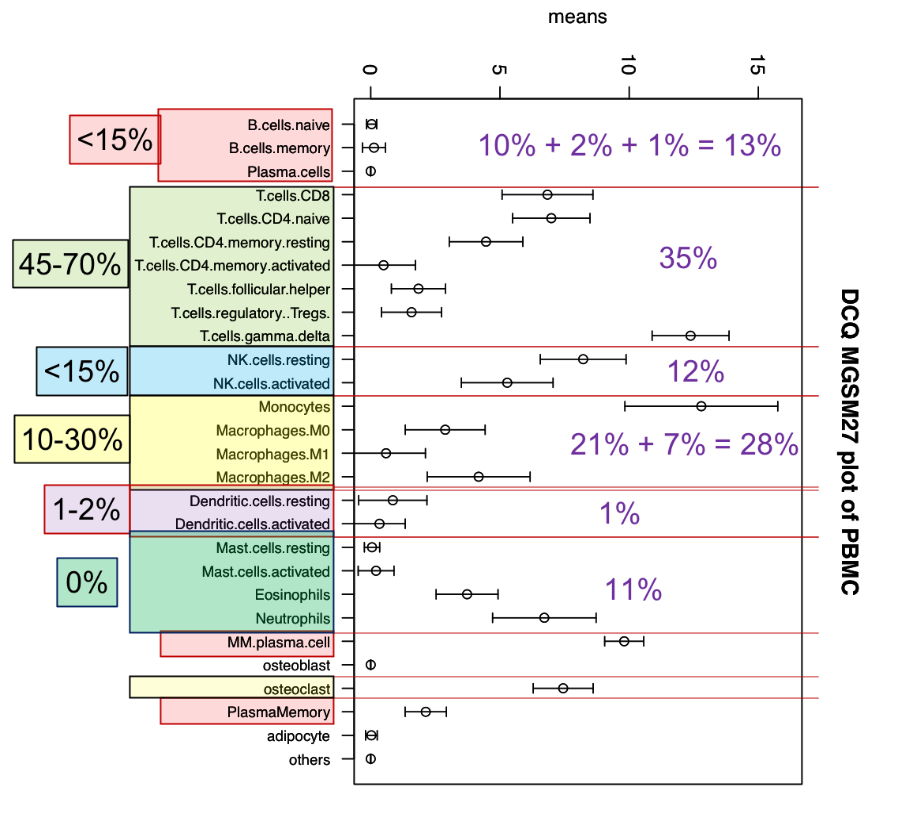


Mean

DCQ MGSM27 plot of PBMC

**H**

| Algorithms | Aspirates  (N = 426), RMSE | WBM  (N = 440), RMSE |
| --- | --- | --- |
| **DCQ [1]** | 10.95 | 14.79 |
| **SVMDECON [2]** | 38.35 | 15.13 |
| **ISOpure [3]** | 23.94 | 20.25 |
| **DSA [4]** | 82.77 | 32.44 |
| **Proportions in admixture [5]** | 30.67 | 13.09 |
| **DeconRNASeq [6]** | 22.94 | 14.88 |

**I**

| Algorithms | Neutrophils, Spearman's ρ | NK cells, Spearman's ρ | Macrophages, Spearman's ρ | T cells, Spearman's ρ |
| --- | --- | --- | --- | --- |
| **DCQ [1]** | 0.909 | 0.515 | 0.7 | 0.399 |
| **DeconRNASeq [6]** | 0.885 | 0.388 | 0.506 | 0.182 |
| **ISOpure [3]** | 0.778 | 0.414 | 0.594 | 0.004 |
| **SVMDECON [2]** | 0.824 | 0.126 | 0.264 | 0.234 |
| **DSA [4]** | 0.931 | 0.649 | NA | 0.324 |
| **Proportions in admixture [5]** | 0.853 | 0.117 | 0.456 | 0.267 |

**References**

1. Altboum Z, Steuerman Y, David E, Barnett-Itzhaki Z, Valadarsky L, Keren-Shaul H, et al. Digital cell quantification identifies global immune cell dynamics during influenza infection. Mol Syst Biol. 2014; 10:720. https://doi.org/10.1002/msb.134947 PMID: 24586061
2. Newman AM, Liu CL, Green MR, Gentles AJ, Feng W, Xu Y, et al*.* Robust enumeration of cell subsets from tissue expression profiles. Nat Methods. 2015; 12:453–7. https://doi.org/10.1038/nmeth.3337 PMID: 25822800
3. Anghel CV, Quan G, Haider S, Nguyen F, Deshwar AG, Morris QD, et al. ISOpureR: an R implementation of a computational purification algorithm of mixed tumour profiles. BMC Bioinformatics. 2015; 16:156. https://doi.org/10.1186/s12859-015-0597-x PMID: 25972088
4. Zhong Y, Wan YW, Pang K, Chow LM, Liu Z. Digital sorting of complex tissues for cell type-specific gene expression profiles. BMC Bioinformatics. 2013; 14:89. https://doi.org/10.1186/1471-2105-14-89 PMID: 23497278
5. Langfelder P, Horvath S. WGCNA: an R package for weighted correlation network analysis. BMC Bioinformatics. 2008; 9:559. https://doi.org/10.1186/1471-2105-9-559 PMID: 19114008
6. Gong T, Szustakowski JD. DeconRNASeq: a statistical framework for deconvolution of heterogeneous tissue samples based on mRNA-Seq data. Bioinformatics. 2013; 29:1083–5. https://doi.org/10.1093/bioinformatics/btt090 PMID: 23428642
