# Supplementary material for: Bone marrow microenvironments that contribute to patient outcomes in newly diagnosed multiple myeloma: A cohort study of patients in the Total Therapy clinical trials
Source: PLoS Med. 2020 Nov 4;17(11):e1003323. doi: 10.1371/journal.pmed.1003323 (PMC7641353; doi:10.1371/journal.pmed.1003323)
Supplement: S2 Fig — (A) Pretreatment PFS for the “low-granulocyte” Cluster 5 (orange) versus the other 4 clusters. (B) Pretreatment OS for the “low-granulocyte” Cluster 5 (orange) versus the other 4 clusters. (C) Postinduction PFS for the “low-granulocyte” Cluster 5 (orange) versus Clusters 1–4 (blue). (D) Postinduction OS for the “low-granulocyte” Cluster 5 (orange) versus Clusters 1–4 (blue). The p-values in the legend compare each cluster to all others with a χ2 test. Note that for (A) and (B) the Cluster 5 estimated mean (rmean) and χ2 p-values are slightly different from that reported in Fig 3. This is due to how having 5 clusters instead of 2 changes the calculation. OS, overall survival; PFS, progression-free survival. (DOCX) [file pmed.1003323.s011.docx]

**S2 Fig. Additional patient outcomes based on microenvironment cluster**

**A**

**
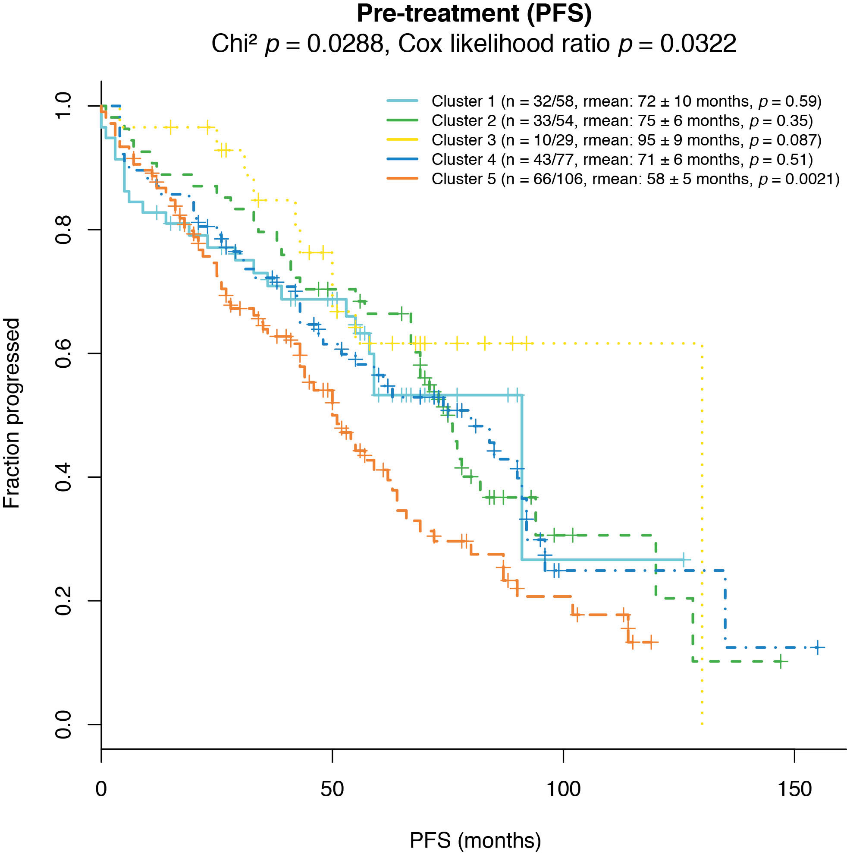
**

Fraction remaining

**B**

**
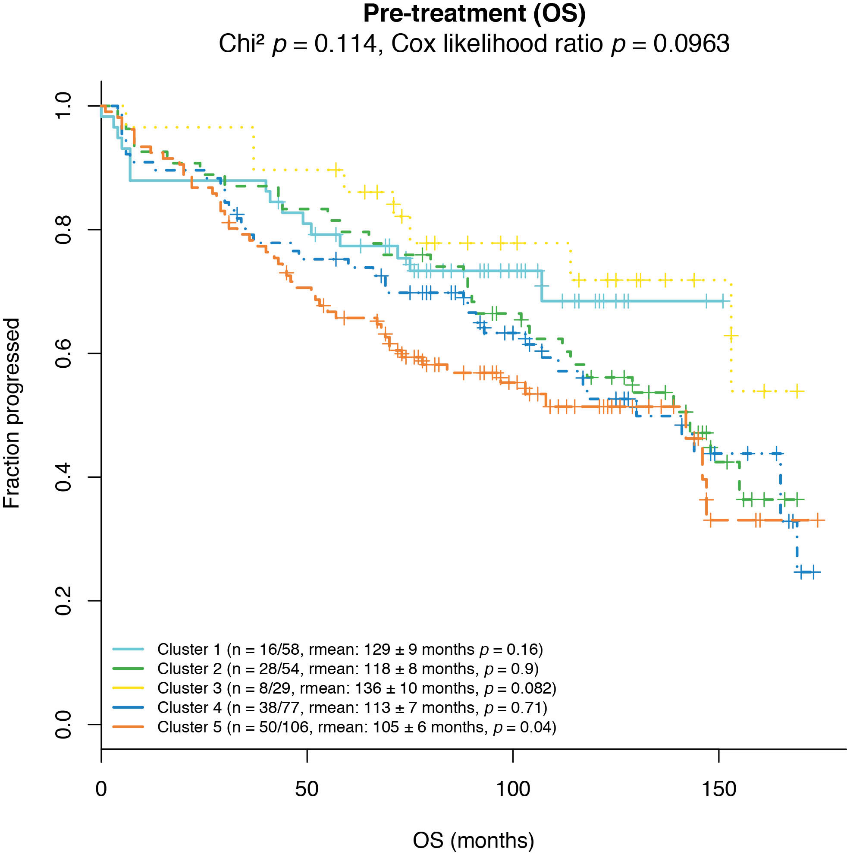
**

Fraction remaining

**C**

**
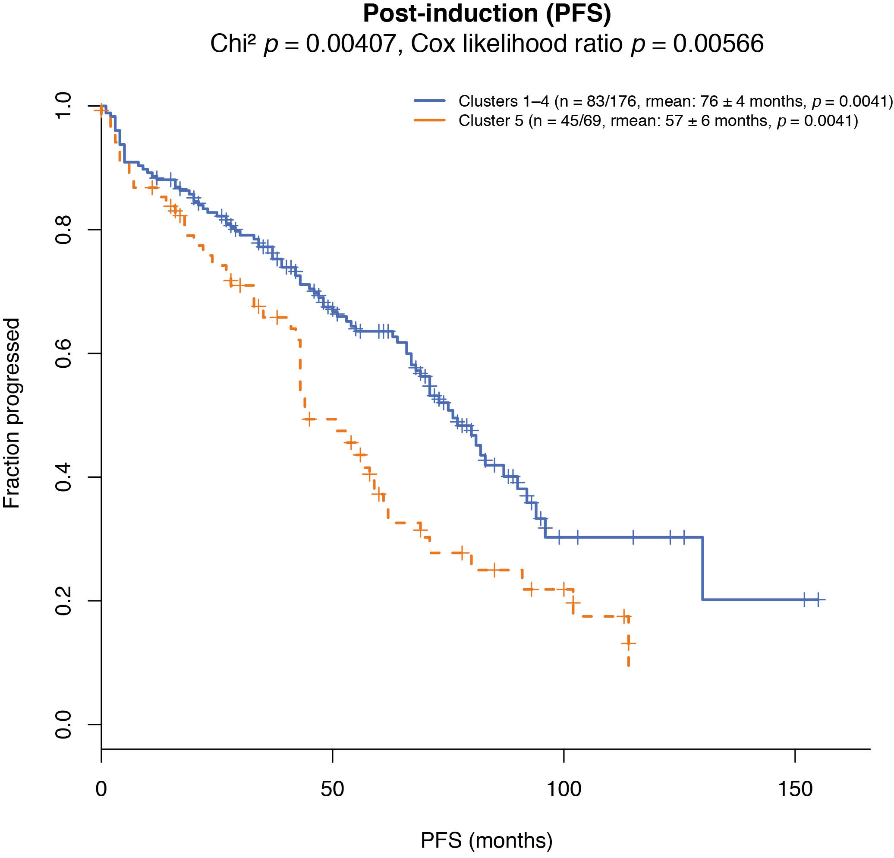
**

Fraction remaining

**D**

**
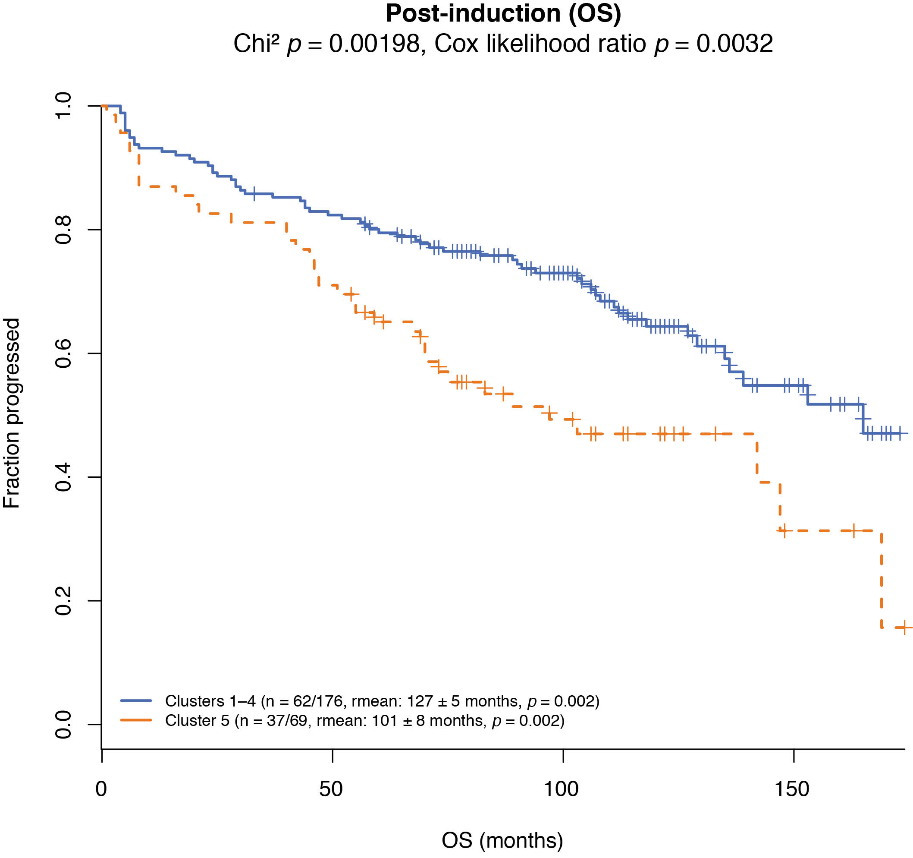
**

Fraction remaining
