# Supplementary material for: Bone marrow microenvironments that contribute to patient outcomes in newly diagnosed multiple myeloma: A cohort study of patients in the Total Therapy clinical trials
Source: PLoS Med. 2020 Nov 4;17(11):e1003323. doi: 10.1371/journal.pmed.1003323 (PMC7641353; doi:10.1371/journal.pmed.1003323)
Supplement: S3 Fig — p-values from Wilcoxon tests for each cell type, relapse samples versus complete remission samples. Population: 17 patients with a sample within 180 days prior to relapse or 30 days after relapse, excluding baseline samples meeting this criterion; 108 samples taken from patients while in complete remission. Rows in yellow pass an FDR cutoff of 0.20; rows in blue pass an FDR cutoff of 0.05. FDR, false discovery rate; NK, natural killer; N/A, not applicable. (DOCX) [file pmed.1003323.s012.docx]

**S3 Fig. Characteristics of patients who will relapse**

| **Factor** | **“Will relapse” direction** | **Wilcoxon *p*-value** | **Wilcoxon FDR** |
| --- | --- | --- | --- |
| Adipocytes | Down | 0.0338 | 0.1014 |
| B cells | – | 0.5505 | 0.8258 |
| CD4 cells | – | 0.6916 | 0.9077 |
| CD8 cells | – | 0.8627 | 0.9109 |
| Dendritic cells | – | 0.9914 | 0.9914 |
| Eosinophils | Down | 0.0388 | 0.1019 |
| Healthy plasma cells | Up | 0.0059 | 0.0415 |
| M0 macrophages | – | 0.5238 | 0.8258 |
| M1 macrophages | – | 0.8675 | 0.9109 |
| M2 macrophages | – | 0.2582 | 0.4929 |
| Mast cells | Down | 0.0067 | 0.0415 |
| Monocytes | Down | 0.0200 | 0.0700 |
| Myeloma | Up | 0.0073 | 0.0415 |
| Neutrophils | Down | 0.0079 | 0.0415 |
| NK cells | – | 0.0501 | 0.1169 |
| Osteoblast | – | 0.8655 | 0.9109 |
| Osteoclast | Down | 0.0185 | 0.0700 |
| Others | – | N/A | N/A |
| T cells gamma/delta | – | 0.2086 | 0.4380 |
| Osteoblast/osteoclast | – | 0.3454 | 0.6045 |
| M1/M2 macrophages | – | 0.6552 | 0.9077 |
| Innate/adaptive cells | – | 0.8515 | 0.9109 |
