# Supplementary figures and images for: Bone marrow microenvironments that contribute to patient outcomes in newly diagnosed multiple myeloma: A cohort study of patients in the Total Therapy clinical trials
Source: PLoS Med. 2020 Nov 4;17(11):e1003323. doi: 10.1371/journal.pmed.1003323 (PMC7641353; doi:10.1371/journal.pmed.1003323)

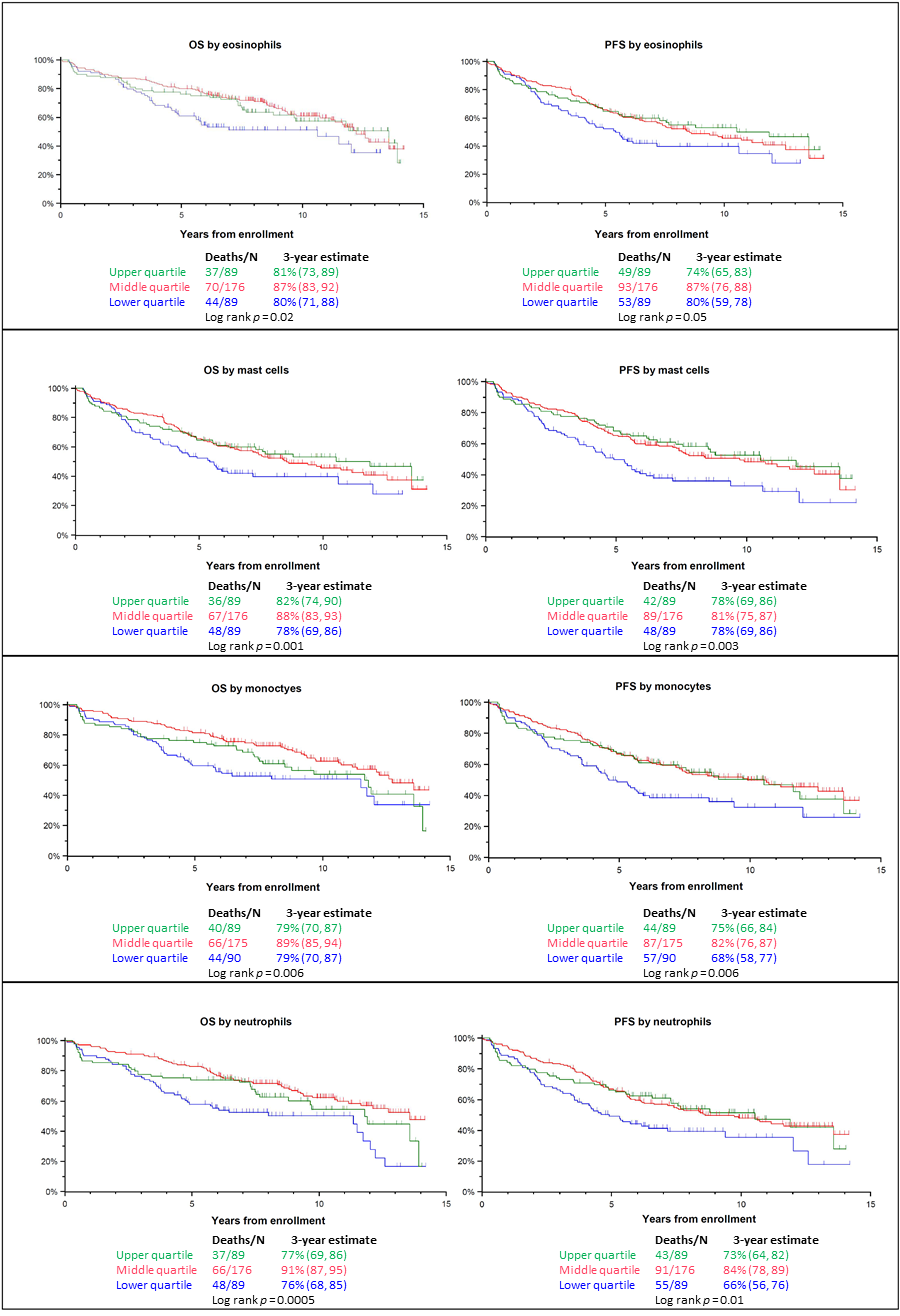
**S4 Fig**

**D**

**C**

**B**

**A**

Supplement: S4 Fig — (A) Eosinophils. (B) Mast cells. (C) Monocytes. (D) Neutrophils. The 2 middle quartiles are combined into a single red line. OS, overall survival; PFS, progression-free survival. (DOCX) [file pmed.1003323.s013.docx]

**S5 Fig.** **Gene-set enrichment by bicluster**
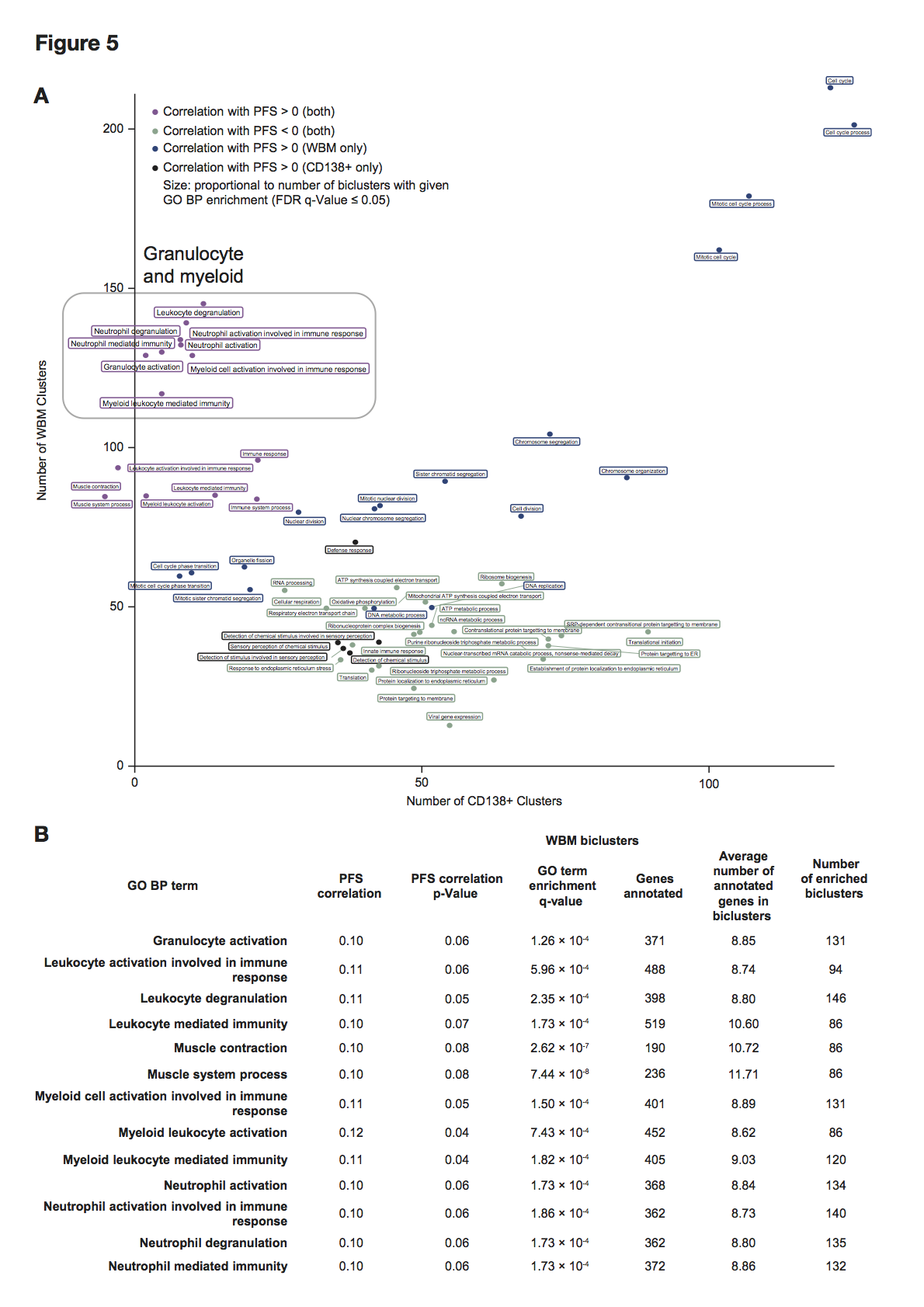


**B**

**A**

Supplement: S5 Fig — As an orthogonal approach to validate the biological relevance of low-granulocyte populations in the generation of a high-risk microenvironment, we constructed patient-specific clusters of coexpressed genes (i.e., biclusters) [31] using cMonkey2 separately on WBM and CD138+-selected samples. cMonkey2 generates gene clusters that are often highly enriched in genes of related function and/or in related biochemical or regulatory pathways [32–34]. We expected biclusters from this analysis to be enriched for genes driven by specific processes in cell types that are transcriptionally related. By contrasting those biclusters between the 2 data sets, we expected to identify processes that were specific to the high-risk microenvironment. (A) cMonkey2 biclusters significantly correlated with patient PFS. Shown are GO terms enriched in cMonkey2 biclusters (out of a maximum 9,258 clusters for CD138+ data and 8,540 clusters for WBM data). The y-axis shows the number of clusters built on the pretreatment WBM samples; the x-axis shows the number of clusters built on CD138+ samples. This analysis resulted in 8,540 (potentially redundant) WBM biclusters and 9,258 CD138+ biclusters, each containing an average of 30.0 and 30.3 genes, respectively, from 187.0 and 253.6 patients, respectively. After performing functional enrichment analysis using the GO terms [35,36], we found that although many common functions (e.g., cell cycle) were enriched in similar numbers of biclusters between the 2 data sets, many more biclusters generated from the WBM data (approximately 130 biclusters, or approximately 1.5% of all biclusters) were enriched for granulocyte- and myeloid-related genes (specifically, activation or degranulation of neutrophils, leukocytes, and/or myeloid cells) than were biclusters generated from the CD138+ data (approximately 15 biclusters, or approximately 0.15%). (B) shows the top GO terms enriched in WBM clusters. The greater number of biclusters enriched for granulocyte-relat [file pmed.1003323.s014.docx]

**S8 Fig.** **Figure 5A with combined mast cells and eosinophils**

**A**

**
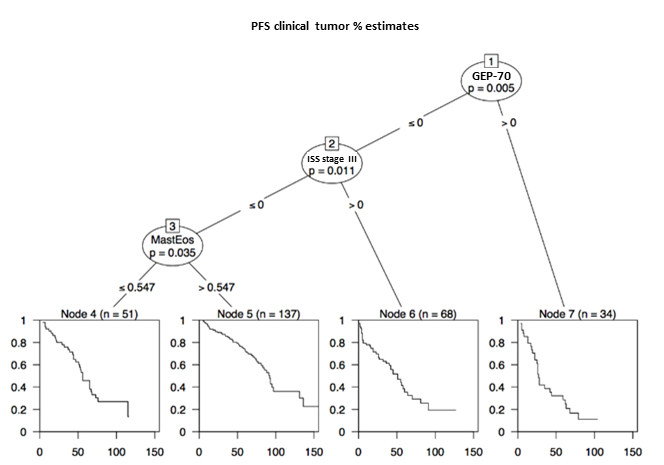
**

**B**

**
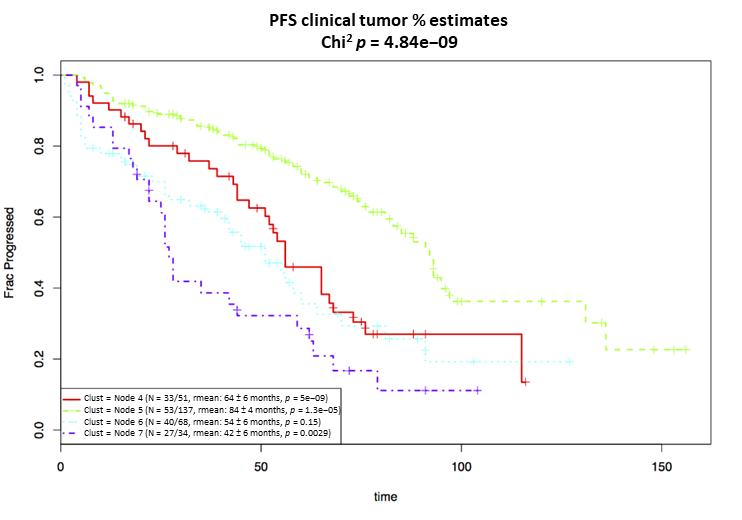
**

Supplement: S8 Fig — (A) Cox proportional hazards conditional inference tree that selected an optimal combination of attributes from the cell types and clinical characteristics for the 290 pretreatment samples with known cytogenetics and tumor burden estimates. MastEos is the sum of mast cells and eosinophils. (B) Survival curves with statistics for the 4 groups identified in S8A Fig. Eos, eosinophils; GEP-70, 70-gene Prognostic Risk Score; ISS3, International Staging System stage III; mo, months; PFS, progression-free survival. (DOCX) [file pmed.1003323.s017.docx]
