# Supplementary material for: Bone marrow microenvironments that contribute to patient outcomes in newly diagnosed multiple myeloma: A cohort study of patients in the Total Therapy clinical trials
Source: PLoS Med. 2020 Nov 4;17(11):e1003323. doi: 10.1371/journal.pmed.1003323 (PMC7641353; doi:10.1371/journal.pmed.1003323)
Supplement: S6 Fig — Patient outcomes as determined by IHC for the 290 patients with IHC samples collected at the same time as samples for microarray. Correlations between deconvolved and proximal pathologist-estimated eosinophils and neutrophils (segmented cells) were 0.25; p = 1.6 × 10^−5^ and 0.36 and p = 2.3 × 10^−10^, respectively. (A) Shown are conditional inference trees and permutation p-values using the pathologist-estimated segmented cell percentages to stratify patients by PFS. (B) Shown are conditional inference trees and permutation p-values using the pathologist-estimated segmented cell percentages to stratify patients by OS. (C) Shown are the same measurements as (A) using pathologist-estimated eosinophil density. (D) Shown are the same measurements as (B) using pathologist-estimated eosinophil density. (E) shows the comparison between CD117 (c-Kit)-labeled cells for 20 samples in which the tumor was CD117– and deconvolved cell estimates and ratios. The first column shows the correlated coefficient with CD117+ cells that morphologically appear to be mature mast cells. The second column shows the p-value associated with the mature mast cell correlations. The third column shows the correlated coefficient with CD117+ cells that morphologically appear to be immature mast cells. The fourth column shows the p-value associated with the immature mast cell correlations. CD, cluster of differentiation; Eos, eosinophils; IHC, immunohistochemistry; NK, natural killer; OS, overall survival; PFS, progression-free survival. (DOCX) [file pmed.1003323.s015.docx]

**S6 Fig. Immunohistochemistry (IHC)**

**A**

**B**


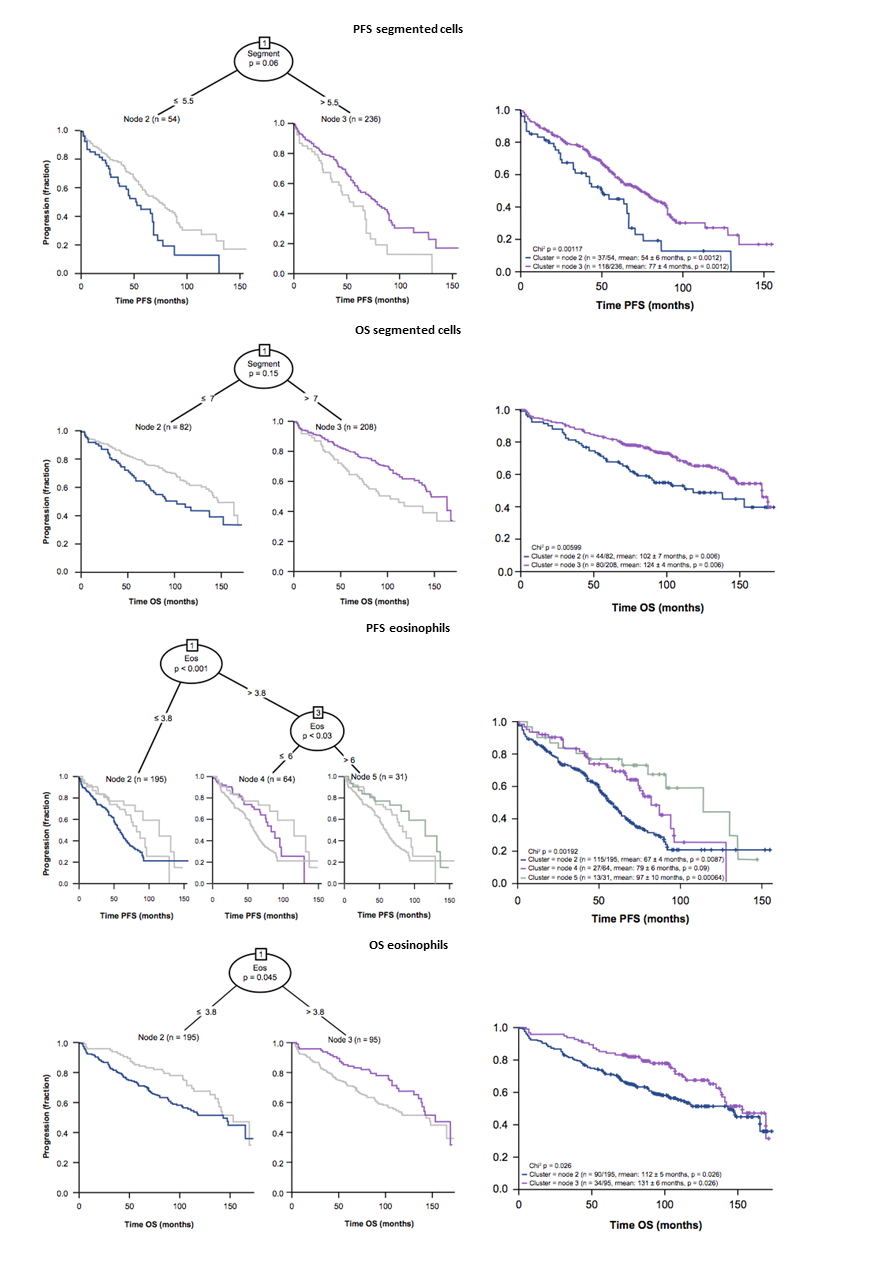


**D**

**C**

**E**

|  | Correlation with mast cells | Mast cell *p-*value | Correlation with immature mast cells | Immature mast cell *p-*value |
| --- | --- | --- | --- | --- |
| Adipocytes | 0.346 | 0.135 | 0.308 | 0.186 |
| B cells | 0.51 | 0.022 | −0.423 | 0.063 |
| CD4 | — | — | — | — |
| CD8 | −0.056 | 0.816 | −0.121 | 0.613 |
| Dendritic cells | 0.219 | 0.353 | 0.475 | 0.034 |
| Eosinophils | −0.022 | 0.927 | 0.188 | 0.427 |
| Healthy plasma cells | 0.053 | 0.825 | −0.397 | 0.083 |
| M0 macrophages | 0.07 | 0.769 | 0.435 | 0.055 |
| M1 macrophages | 0.012 | 0.959 | 0.163 | 0.492 |
| M2 macrophages | 0.054 | 0.82 | 0.419 | 0.066 |
| Mast cells | −0.127 | 0.594 | 0.319 | 0.171 |
| Monocytes | −0.159 | 0.504 | 0.489 | 0.029 |
| Myeloma | −0.106 | 0.657 | −0.474 | 0.035 |
| Neutrophils | −0.181 | 0.444 | 0.449 | 0.047 |
| NK cells | −0.08 | 0.737 | 0.433 | 0.056 |
| Osteoblasts | 0.411 | 0.072 | −0.174 | 0.464 |
| Osteoclasts | 0.197 | 0.404 | 0.602 | 0.005 |
| Others | — | — | — | — |
| Gamma delta T cells | 0.081 | 0.94 | 0.354 | 0.126 |
| Osteoblasts/osteoclasts | 0.423 | 0.063 | −0.528 | 0.017 |
| M1 macrophages/M2 macrophages | −0.015 | 0.95 | 0.138 | 0.561 |
| Innate cells/adaptive cells | −0.437 | 0.054 | −0.207 | 0.381 |
