# Supplementary material for: Bone marrow microenvironments that contribute to patient outcomes in newly diagnosed multiple myeloma: A cohort study of patients in the Total Therapy clinical trials
Source: PLoS Med. 2020 Nov 4;17(11):e1003323. doi: 10.1371/journal.pmed.1003323 (PMC7641353; doi:10.1371/journal.pmed.1003323)
Supplement: S7 Fig — p-values were calculated using an FDR-corrected Wilcoxon signed-rank test. DNA, deoxyribonucleic acid; FDR, false discovery rate. (DOCX) [file pmed.1003323.s016.docx]

**S7 Fig. Top differentially expressed tumor genes as segmented by deconvolved mast-cell quartiles**

| **Gene** | **Log_2_ (mast low/mast high)** | **Wilcoxon**  **FDR**  **(n = 328)** | **Description** |
| --- | --- | --- | --- |
| *FANCL* | 0.77 | 8.13 × 10^-7^ | (FA Complementation Group L) Ubiquitin-protein ligase |
| *ZNF267* | 0.71 | 2.06 × 10^-6^ | (Zinc Finger Protein 267) Zinc finger protein |
| *MITD1* | 0.66 | 4.87 × 10^-6^ | (Microtubule interacting and trafficking domain) Cell division |
| *OTUD6B* | 0.66 | 1.26 × 10^-5^ | (OTU Domain Containing 6B) Functional deubiquitinating enzyme, tumor proliferation |
| *SLC25A43* | 0.83 | 1.80 × 10^-5^ | (Solute Carrier Family 25 Member 43) Mitochondrial solute carrier |
| *SPPL2A* | 0.67 | 2.23 × 10^-5^ | (Signal peptide peptidase-like protease) Cleaves tumor necrosis factor-alpha |
| *NET1* | 0.68 | 2.60 × 10^-5^ | (Guanine nucleotide regulatory protein) Guanine nucleotide exchange factor, breast cancer associated |
| *NIPSNAP3A* | 0.79 | 3.01 × 10^-5^ | Not annotated |
| *LAMP3* | 0.76 | 8.90 × 10^-5^ | (Lysosomal Associated Membrane Protein 3) Lysosomal membrane protein, exclusively in mature dendritic cells |
| *MAD2L1* | 0.69 | 1.85 × 10^-5^ | (Mitotic Arrest Deficient 2 Like 1) Mitotic spindle assembly checkpoint protein |
| *NDC80* | 0.69 | 2.20 × 10^-4^ | Kinetochore protein NDC80 homolog |
| *ZNF367* | 0.67 | 2.54 × 10^-4^ | (Zinc Finger Protein 367) Zinc finger protein, expressed in fetal erythroid tissue and tumors |
| *TMA16* | 0.72 | 3.33 × 10^-4^ | (Translation machinery associated) |
| *IFIT1* | 0.99 | 3.82 × 10^-4^ | (Interferon-induced protein with tetratricopeptide repeats 1) |
| *PLSCR1* | 0.75 | 4.02 × 10^-4^ | (Phospholipid scramblase 1) Interferon response |
| *HGF* | 0.95 | 5.86 × 10^-4^ | (Hepatocyte growth factor) |
| *PRG2* | −0.75 | 7.52 × 10^-4^ | (Proteoglycan 2) Eosinophil major basic protein, mast cell activation |
| *ISG15* | 0.75 | 9.16 × 10^-4^ | (Interferon-stimulated gene) Type I lipopolysaccharide, stimulated gene, ubiquitin cross-reactive. Pancreatic adenocarcinoma tumor-associated macrophages |
| *GBP1* | 0.76 | 9.54 × 10^-4^ | (Guanylate Binding Protein 1) Interferon-induced guanylate-binding, pathogen response mediator |
| *SCYL2* | 0.71 | 1.17 × 10^-3^ | (SCY1 Like Pseudokinase 2) Pseudokinase, *WNT* signaling pathway |
| *CLIC2* | 0.78 | 1.46 × 10^-3^ | (Chloride intracellular channel protein) X-linked |
| *TRAT1* | 1.05 | 1.66 × 10^-3^ | (T cell receptor-associated adaptor 1) Affects T cell function |
| *NECTIN3* | 0.74 | 1.73 × 10^-3^ | (Nectin cell adhesion molecule 3) Inhibits mast-cell adhesion? |
| *IFIT3* | 0.74 | 2.54 × 10^-3^ | (Interferon-induced protein with tetratricopeptide repeats 3) |
| *IFI6* | 0.69 | 3.49 × 10^-3^ | (Interferon Alpha Inducible Protein 6) Apoptosis-related |
| *VCAM1* | −0.78 | 4.39 × 10^-3^ | (Vascular cell adhesion molecule 1) Adhesion protein for circulating mast cell precursors |
| *APOBEC3B* | 0.76 | 4.65 × 10^-3^ | Probable DNA dC->dU-editing enzyme, cell cycle |
| *TMEM255A* | 0.93 | 4.83 × 10^-3^ | (Transmembrane Protein 255A) Nuclear envelope |
| *IFI44L* | 0.83 | 7.84 × 10^-3^ | (Interferon-induced protein 44 Like) Estrogen-induced, lupus-related |
| *FAM133A* | 0.72 | 8.19 × 10^-3^ | (Family with sequence similarity 133 member A) Brain and testes, X-linked. Multiple myeloma marker gene |
| *RASSF6* | 0.75 | 1.59 × 10^-2^ | (Ras association domain family member 6) Highly conserved tumor suppressor network |
| *SMAD1* | 0.85 | 1.65 × 10^-2^ | (SMAD Family Member 1) Signal transducers and transcriptional modulators |
