# Supplementary material for: Bone marrow microenvironments that contribute to patient outcomes in newly diagnosed multiple myeloma: A cohort study of patients in the Total Therapy clinical trials
Source: PLoS Med. 2020 Nov 4;17(11):e1003323. doi: 10.1371/journal.pmed.1003323 (PMC7641353; doi:10.1371/journal.pmed.1003323)
Supplement: S9 Fig — Overlapping ellipses generated by ggbiplot show no separation of data by batch or site. (DOCX) [file pmed.1003323.s018.docx]

**S9 Fig.** **Principal component analysis plot of all microarrays that passed quality control by process site and batch**

Groups
