# Supplementary material for: Bone marrow microenvironments that contribute to patient outcomes in newly diagnosed multiple myeloma: A cohort study of patients in the Total Therapy clinical trials
Source: PLoS Med. 2020 Nov 4;17(11):e1003323. doi: 10.1371/journal.pmed.1003323 (PMC7641353; doi:10.1371/journal.pmed.1003323)
Supplement: S10 Fig — “1st Samp” shows the distribution for patients who had only 1 sample (black). For patients with multiple samples in a single treatment phase, their sequential time points are colored red (first), light blue (second), dark blue (third), green (fourth), and orange (fifth). (DOCX) [file pmed.1003323.s019.docx]

**S10 Fig. Violin plots of multi-sampled patients across treatment**

Post-consolidation

Post-transplant

Post-induction

Observation of total observations per individual

Days after baseline sample

Post-maintenance
